# Association between complete blood count-derived inflammatory markers and the risk of frailty and mortality in middle-aged and older adults

**DOI:** 10.3389/fpubh.2024.1427546

**Published:** 2024-07-31

**Authors:** Yu Tang, Ying Zhai, Wenjing Song, Tengxiao Zhu, Zichen Xu, Luqing Jiang, Lei Li, Daoqin Liu, Qiwen Wu

**Affiliations:** ^1^Clinical Laboratory, The First Affiliated Hospital of Wannan Medical College, Wuhu, China; ^2^Department of Kidney Medicine, The First Affiliated Hospital of Wannan Medical College, Wuhu, China

**Keywords:** CBC-derived inflammatory markers, frailty, mortality, RSF, NHANES

## Abstract

**Objective:**

This study aimed to evaluate the association between six complete blood count (CBC)-derived inflammatory markers [neutrophil-to-lymphocyte ratio (NLR), monocyte-to-lymphocyte ratio (MLR), platelet-to-lymphocyte ratio (PLR), systemic immune-inflammatory index (SII), systemic inflammatory response index (SIRI), and pan-immune inflammation value (PIV)] and the risk of frailty and mortality.

**Methods:**

Data were obtained from the National Health and Nutrition Examination Survey (NHANES) 1999–2018. Mortality was identified using the National Death Index until December 31, 2019. Multiple logistic regression analysis was conducted to evaluate the association between six CBC-derived inflammatory markers and frailty. The Cox regression model assessed the association between six CBC-derived inflammatory markers and mortality in frail populations. Restricted cubic spline (RCS) was used to visualize the association of the six CBC-derived inflammatory markers with mortality risk. The predictive value of CBC-derived inflammatory markers for mortality was further assessed using a random survival forest (RSF) approach.

**Results:**

This study analyzed data from a total of 16,705 middle-aged and older participants. Among them, 6,503 participants were frail, with a mortality rate of 41.47%. Multiple logistic regression analysis showed that NLR, MLR, PLR, SII, SIRI, and PIV were positively associated with frailty risk. The Cox regression model revealed that participants in the highest quartile had a significantly increased risk of death compared to those in the lowest quartile: NLR (HR = 1.73, 95% CI:1.54, 1.94), MLR (HR = 1.71, 95% CI:1.51, 1.93), PLR (HR = 1.28, 95%CI: 1.15, 1.43), SII (HR = 1.50, 95%CI:1.34, 1.68), SIRI (HR = 1.88, CI 95%:1.67, 2.12), PIV (HR = 1.55, 95%CI:1.38, 1.73). Random survival forest (RSF) analyses demonstrated that MLR had the highest predictive value for mortality risk middle-aged and older adult frail participants.

**Conclusion:**

The results suggest that CBC-derived inflammatory markers are associated with a higher risk of frailty as well as mortality in the middle and old-aged population of the United States.

## 1 Introduction

Frailty represents a burgeoning global health concern (1). Studies have shown that frailty is prevalent in the aging population, and its incidence escalates with age (2). This age-related syndrome disrupts bodily homeostasis, resulting in the loss of physiological functions and immune system abnormalities (3, 4). Frail patients exhibit diminished resilience to both external and internal stressors, leading to acute health fluctuations in response to minor perturbations (5). Frail older adults face substantially heightened susceptibility to various adverse outcomes, such as falls, disability, hospitalization, and mortality (6).

Studies have shown that even in the absence of infection, frail older adults develop chronic low-grade inflammation over time (7, 8). This chronic inflammation, which leaves the organism in a protracted state of inflammation, ultimately accumulates, and exacerbates damage (9). Chronic inflammation, characterized by elevated levels of inflammatory cytokines, can play a role in the development of frailty, and is strongly associated with frailty and premature death (10, 11). With the understanding of the mechanisms of frailty, there is a need for improved markers to assess the prognosis of frailty.

CBC-derived inflammatory markers are composed of neutrophils, lymphocytes, platelets, and monocytes. These markers are utilized in the predictive assessment of various inflammation-related diseases (12, 13). Recent studies have shown that the neutrophil-to-lymphocyte ratio (NLR), platelet-to-lymphocyte ratio (PLR), and pan-immuno-inflammatory value (PIV) can serve as indicators of inflammation associated with frailty (14, 15). Additionally, the Systemic Immunoinflammatory Index (SII), derived from CBC, has been shown to have the potential to predict the risk of frailty and mortality in middle-aged and older adult frail patients (16). However, the association between other CBC-derived inflammatory markers and the risk of frailty as well as mortality in frail patients remains incompletely assessed. Therefore, this study aimed to investigate the prognostic significance of inflammatory markers derived from complete blood counts in frail individuals.

## 2 Materials and methods

### 2.1 Study population

All participants in the National Health and Nutrition Examination Survey (NHANES) provided written consent to voluntarily participate in the study, and the NHANES study protocol was approved by the Research Ethics Review Board of the National Center for Health Statistics. We examined participant information for 10 cycles from 1999 to 2018. The study excluded participants aged below 45 years, with unavailable CBC data, and lacking follow-up information. A frailty index (FI) was utilized to categorize the remaining participants into frail participants (*n* = 6,503) and non-frail participants (*n* = 10,202), as depicted in Supplementary Figure S1.

Referring to previous related studies using the NHANES, a set of 36 variables was utilized to construct the FI, encompassing domains of disease, physical functioning, and laboratory tests (17, 18). Each variable was scored as either dichotomous (taking 0 or 1) or continuous (ranging from 0 to 1). A higher score for each item indicates greater impairment. The total score for all items is divided by 36 (the total number of items) to derive the FI. Finally, participants were categorized as non-frail (FI < 0.25) and frail (FI ≥ 0.25) according to their FI scores (19, 20). Supplementary Table S1 demonstrates the deficiency items and scoring criteria for inclusion in the FI.

### 2.2 Definition of CBC-derived inflammatory markers

The methods used to derive complete blood count (CBC) parameters utilize the Beckman Coulter method of counting and sizing, along with an automatic diluting and mixing device for sample processing. Indicators were computed using the following formulas: MLR = monocytes/lymphocytes, NLR = neutrophils/lymphocytes, PLR = platelets/lymphocytes, SII = platelets × neutrophils/lymphocytes, SIRI = neutrophils × monocytes/lymphocytes, PIV = neutrophils × monocytes × platelets/lymphocytes (12, 15). Since platelets are available only as absolute counts and not as percentages, to ensure the uniformity of the data, all CBC-derived inflammatory indices in this study were obtained by calculating the absolute counts of complete blood cells.

### 2.3 Covariates

To assess the impact of underlying factors, several covariates obtained through questionnaires and physical examinations are utilized. These variables encompass gender, age, race, education, marital status, family poverty income ratio (PIR), body mass index (BMI), smoking status, and drinking status. Race was classified as Mexican American, Other Hispanic, Non-Hispanic White, Non-Hispanic Black, or Other Race. Educational level is classified into below high school, high school, or equivalent, and above high school. Marital status is divided into two categories: married (including cohabitation with a partner) and unmarried (including widowed, separated, and divorced). Smoking status was classified into three categories: never smokers (<100 cigarettes before the survey), former smokers (>100 cigarettes before the survey, currently quitting), and current smokers (>100 cigarettes before the survey, currently smoking). Drinking status was divided into two categories: heavy drinker (≥2 drinks/day for men and ≥1 drink/day for women) and non-heavy drinker (<2 drinks/day for men and <1 drink/day for women) (21).

### 2.4 Assessment of mortality

This study considers all-cause mortality as the endpoint, with causes of death encompassing heart disease, malignancy, chronic lower respiratory tract disease, cerebrovascular disease, and other causes. Survival time was determined from the NHANES mobile examination center (MEC) date, with follow-up ending on December 31, 2019. Death data were acquired from the National Center for Health Statistics.

### 2.5 Statistical analysis

Participants’ basic characteristics were expressed as median (interquartile range [IQR]) for continuous variables and as number (percentage) for categorical variables. The Kruskal-Wallis rank sum test was used for comparisons of continuous variables, and the chi-square test was used for comparisons of categorical variables. For missing data on covariates, the MissForest R package was employed to fill in.

The CBC-derived inflammatory markers were divided into quartiles, with the lowest quartile serving as the reference. Multivariate logistic regression models were employed to assess the odds ratios (OR) and 95% confidence intervals (95% CI) for the association between CBC-derived inflammatory markers and frailty. Hazard ratios (HR) and 95% confidence intervals (95% CI) for all-cause mortality in frail patients were determined using COX regression analysis. Model 2 adjusted for age, gender, and race. Model 3 adjusted for age, gender, race, marital status, education level, family poverty income ratio, body mass index, smoking status, and alcohol status. To further explore the relationship between CBC-derived inflammatory markers and the risk of death in frail patients, restricted cubic spline regression analyses were additionally conducted. The knots were placed at each exposure variable’s 5th, 35th, 65th, and 95th percentiles.

Additionally, the odds ratio (OR) and 95% confidence interval (95% CI) between CBC parameters and frailty were calculated using the same method. The hazard ratio (HR) and 95% confidence interval (95% CI) between CBC parameters and death in frail patients were further analyzed. A random survival forest approach was employed to compare the predictive value of CBC-derived inflammatory markers and CBC parameters for all-cause mortality in frail patients.

Sensitivity analyses were conducted to evaluate the robustness of the results. Due to the controversy surrounding the FI critical value defining the frailty state, an alternative critical value was used to redefine the frailty state. Frailty was defined as FI > 0.21, while non-frailty was defined as FI ≤ 0.21. Consequently, the FI was reconstructed after excluding platelet counts from the original FI, and the main analyses were conducted. R (version 4.3.3) performed all analyses.

## 3 Results

### 3.1 Characteristics of the study population

Table 1 displays the baseline characteristics of middle-aged and older adult populations with frailty in NHANES 1999–2018. Among the 16,705 middle-aged and older adult participants included, the median age 67 (61.75) years, with 49.40% being male. Among them, 6,503 (38.93%) were identified as frail, the median age 69 (61.78) years, with 45.19% being male. Compared to non-frail participants, frail participants were more likely to be older, non-Hispanic black females, have lower levels of education and income, be unmarried, smoke, consume alcohol lightly, and have a higher BMI (*p* < 0.05). Neutrophil, monocyte and lymphocyte counts were significantly higher and platelet counts were significantly lower in frail patients (*p* < 0.05). Among all CBC-derived inflammatory markers, NLR, MLR, SII, SIRI, and PIV showed significant differences between frail and non-frail participants.

**Table 1:** Baseline characteristics of middle-aged and older adult populations with frailty.

Variable	Total (*N* = 16,705)	Non-frail (*N* = 10,202)	Frail (*N* = 6,503)	*p*-value
Age (years)	67.00 (61.00, 75.00)	66.00 (61.00, 73.00)	69.00 (61.00, 78.00)	<0.001
Gender				<0.001
Male	8,252 (49.40%)	5,313 (52.08%)	2,939 (45.19%)	
Female	8,453 (50.60%)	4,889 (47.92%)	3,564 (54.81%)	
Race				<0.001
Mexican American	2,405 (14.40%)	1,477 (14.48%)	928 (14.27%)	
Other Hispanic	1,386 (8.30%)	862 (8.45%)	524 (8.06%)	
Non-Hispanic White	8,529 (51.06%)	5,284 (51.79%)	3,245 (49.90%)	
Non-Hispanic Black	3,231 (19.34%)	1,840 (18.04%)	1,391 (21.39%)	
Other race	1,154 (6.91%)	739 (7.24%)	415 (6.38%)	
Education level				<0.001
Below high school	5,501 (32.93%)	2,925 (28.67%)	2,576 (39.61%)	
High school	3,954 (23.67%)	2,382 (23.35%)	1,572 (24.17%)	
Above high school	7,250 (43.40%)	4,895 (47.98%)	2,355 (36.21%)	
Marital status				<0.001
Unmarried	6,971 (41.73%)	3,783 (37.08%)	3,188 (49.02%)	
Married	9,734 (58.27%)	6,419 (62.92%)	3,315 (50.98%)	
Family PIR	2.04 (1.18,3.62)	2.39 (1.34,4.13)	1.61 (1.01,2.75)	<0.001
Smoking status				<0.001
Never smoker	7,853 (47.01%)	5,029 (49.29%)	2,824 (43.43%)	
Former smoker	6,109 (36.57%)	3,602 (35.31%)	2,507 (38.55%)	
Current smoker	2,743 (16.42%)	1,571 (15.40%)	1,172 (18.02%)	
Drinking status				<0.001
Non-heavy drinker	13,627 (81.57%)	8,150 (79.89%)	5,477 (84.22%)	
Heavy drinker	3,078 (18.43%)	2052 (20.11%)	1,026 (15.78%)	
BMI (Kg/m^2^)	28.30 (25.00, 32.36)	27.80 (24.70, 31.34)	29.40 (25.62, 34.24)	<0.001
Neutrophils (10^3^/μL)	4.00 (3.10, 5.10)	3.90 (3.00, 4.80)	4.30 (3.30, 5.40)	<0.001
Monocyte (10^3^/μL)	0.60 (0.40, 0.70)	0.50 (0.40, 0.70)	0.60 (0.50, 0.70)	<0.001
Lymphocyte (10^3^/μL)	1.90 (1.50, 2.40)	1.90 (1.50, 2.40)	1.90 (1.50, 2.40)	0.015
Platelet (10^3^/μL)	232.00 (195.00, 276.00)	233.00 (197.00, 275.00)	232.00 (190.00, 279.00)	0.028
NLR	2.06 (1.52, 2.82)	2.00 (1.48, 2.67)	2.22 (1.60, 3.08)	<0.001
MLR	0.29 (0.22, 0.38)	0.28 (0.21, 0.36)	0.30 (0.22, 0.40)	<0.001
PLR	120.69 (94.14, 156.07)	120.80 (95.29, 154.38)	120.53 (91.97, 159.09)	0.931
SII (10^3^/μL)	477.83 (336.00, 684.00)	461.16 (328.25, 647.31)	508.30 (347.49, 745.32)	<0.001
SIRI (10^3^/μL)	1.13 (0.76, 1.69)	1.06 (0.72, 1.56)	1.27 (0.82, 1.91)	<0.001
PIV (10^6^/μL)	259.92 (166.44, 409.60)	243.42 (159.39, 381.25)	290.00 (179.25, 455.91)	<0.001

Family PIR, Family poverty income ratio; NLR, neutrophil-to-lymphocyte ratio; PLR, platelet-to-lymphocyte ratio; MLR, monocyte-to-lymphocyte ratio; SIRI, systemic inflammatory response index; SII, systemic immune-inflammation index; PIV, pan-immune inflammation value; CBC, complete blood cell. The continuous variables are expressed as medians (interquartile spacing). Categorical variables are presented as numbers (percentages). N reflects the study sample.

### 3.2 Association between CBC-derived inflammatory markers and frailty risk

The correlations between CBC-derived inflammatory markers and frailty are presented in Table 2. We found that NLR, MLR, SII, SIRI, and PIV showed positive associations with the risk of frailty in the crude model. The results remained statistically significant after adjusting for gender, age, and race.

**Table 2:** Associations between CBC-derived inflammatory markers and frailty risk in middle-aged and older adult populations.

	Crude		Model 1		Model 2	
	OR (95%CI)	*p* value	OR (95%CI)	*p* value	OR (95%CI)	*p* value
**NLR**						
Quartile1	1 (Reference)		1 (Reference)		1 (Reference)	
Quartile2	1.01 (0.92, 1.11)	0.8317	1.08 (0.98, 1.18)	0.1242	1.07 (0.97, 1.18)	0.1610
Quartile3	1.19 (1.09, 1.30)	0.0001	1.31 (1.19, 1.43)	<0.0001	1.26 (1.15, 1.39)	0.0001
Quartile4	1.80 (1.65, 1.96)	<0.0001	2.01 (1.84, 2.21)	<0.0001	1.96 (1.77, 2.16)	<0.0001
P for trend	1.30 (1.26, 1.35)	<0.0001	1.36 (1.31, 1.42)	<0.0001	1.34 (1.29, 1.40)	<0.0001
**MLR**						
Quartile1	1 (Reference)		1 (Reference)		1 (Reference)	
Quartile2	0.93 (0.85, 1.02)	0.1209	0.98 (0.89, 1.07)	0.6382	1.04 (0.94, 1.14)	0.4343
Quartile3	1.07 (0.98, 1.17)	0.1190	1.16 (1.06, 1.27)	0.0017	1.23 (1.11, 1.35)	<0.0001
Quartile4	1.48 (1.36, 1.62)	<0.0001	1.65 (1.50, 1.82)	<0.0001	1.81 (1.64, 2.01)	<0.0001
P for trend	5.00 (3.69, 6.78)	<0.0001	7.33 (5.25, 10.23)	<0.0001	9.89 (6.96, 14.04)	<0.0001
**PLR**						
Quartile1	1 (Reference)		1 (Reference)		1 (Reference)	
Quartile2	0.83 (0.76, 0.90)	<0.0001	0.81 (0.74, 0.89)	<0.0001	0.86 (0.78, 0.94)	0.0013
Quartile3	0.82 (0.75, 0.89)	<0.0001	0.81 (0.74, 0.88)	<0.0001	0.91 (0.83, 0.99)	0.0499
Quartile4	0.99 (0.91, 1.08)	0.8343	0.95 (0.87, 1.04)	0.2517	1.13 (1.03, 1.24)	0.0115
P for trend	1.00 (1.00, 1.00)	0.5243	1.00 (1.00, 1.00)	0.7798	1.00 (1.00, 1.00)	0.0005
**SII**						
Quartile1	1 (Reference)		1 (Reference)		1 (Reference)	
Quartile2	0.95 (0.87, 1.04)	0.2654	0.98 (0.90, 1.08)	0.7040	0.95 (0.86, 1.05)	0.2928
Quartile3	1.10 (1.01, 1.20)	0.0312	1.15 (1.05, 1.26)	0.0033	1.09 (0.99, 1.20)	0.0646
Quartile4	1.57 (1.44, 1.72)	<0.0001	1.66 (1.51, 1.82)	<0.0001	1.56 (1.42, 1.71)	<0.0001
P for trend	1.00 (1.00, 1.00)	<0.0001	1.00 (1.00, 1.00)	<0.0001	1.00 (1.00, 1.00)	<0.0001
**SIRI**						
Quartile1	1 (Reference)		1 (Reference)		1 (Reference)	
Quartile2	1.03 (0.94, 1.13)	0.4997	1.12 (1.02, 1.23)	0.0143	1.06 (0.96, 1.17)	0.2503
Quartile3	1.42 (1.30, 1.55)	<0.0001	1.62 (1.48, 1.78)	<0.0001	1.49 (1.35, 1.64)	<0.0001
Quartile4	1.95 (1.79, 2.13)	<0.0001	2.32 (2.11, 2.55)	<0.0001	2.03 (1.84, 2.25)	<0.0001
P for trend	1.51 (1.44, 1.59)	<0.0001	1.65 (1.57, 1.74)	<0.0001	1.54 (1.46, 1.63)	<0.0001
**PIV**						
Quartile1	1 (Reference)		1 (Reference)		1 (Reference)	
Quartile2	1.04 (0.95, 1.14)	0.3685	1.09 (1.00, 1.20)	0.0614	1.03 (0.94, 1.14)	0.4839
Quartile3	1.30 (1.19, 1.42)	<0.0001	1.40 (1.27, 1.53)	<0.0001	1.28 (1.17, 1.41)	<0.0001
Quartile4	1.76 (1.62, 1.93)	<0.0001	1.94 (1.77, 2.13)	<0.0001	1.71 (1.55, 1.89)	<0.0001
P for trend	1.00 (1.00, 1.00)	<0.0001	1.00 (1.00, 1.00)	<0.0001	1.00 (1.00, 1.00)	<0.0001

Data are presented as OR (95% CI). Crude adjusts for none. Model 1 adjust for age, gender, and race. Model 2 adjust for Model 1 plus marital status, education level, family poverty income ratio, body mass index, smoking status, and alcohol status.

In Model 2, higher levels of NLR, MLR, PLR, SII, SIRI, and PIV were found to be associated with an increased risk of frailty. Using the lowest quartile as a reference, the odds ratios (OR) and 95% confidence intervals (95% CI) at the highest quartile were NLR (OR = 1.96, 95% CI: 1.77, 2.16), MLR (OR = 1.81, 95% CI: 1.64, 2.01), PLR (OR = 1.13, 95% CI:1.03, 1.24), SII (OR = 1.56, 95% CI:1.42, 1.71), SIRI (OR = 2.03, 95% CI:1.84, 2.25), PIV (OR = 1.71, 95% CI:1.55, 1.89). Notably, lower levels of PLR were nevertheless associated with a decreased frailty risk. Compared with the lowest quartile, the odds ratios for participants in quartile 2 and quartile 3 were (OR = 0.86, 95% CI:0.78, 0.94) and (OR = 0.91, 95% CI:0.83, 0.99), respectively.

Additionally, the relationship between CBC parameters and the risk of frailty was analyzed (Supplementary Table S2). After adjusting for all confounders, we found that higher levels of neutrophil and monocyte counts were positively associated with the risk of frailty, while lymphocyte and platelet counts were negatively associated with the risk of frailty, with statistically significant results (*p* < 0.05).

### 3.3 Relationship between CBC-derived inflammatory markers and risk of death

The median follow-up period was 82.3 months (range: 1–249 months), with 2,697 deaths (41.47%). CBC-derived inflammatory markers (NLR, MLR, PLR, SII, SIRI, and PIV) exhibited a significantly higher risk of death in frail patients in the highest quartile compared to the lowest quartile. In model 2, the hazard ratios (HR) and 95% confidence intervals (95% CI) of death for the CBC-derived inflammatory markers were as follows: NLR (HR = 1.73, 95% CI: 1.54, 1.94), MLR (HR = 1.71, 95% CI: 1.51, 1.93), PLR (HR = 1.28, CI: 1.15, 1.43), SII (HR = 1.50, 95% CI:1.34, 1.68), SIRI (HR = 1.88, 95% CI: 1.67, 2.12), PIV (HR = 1.55, 95% CI: 1.38, 1.73) (Table 3).

**Table 3:** Associations of CBC-derived inflammatory markers with mortality in middle-aged and older adult populations with frailty.

	Crude		Model 1		Model 2	
	HR (95%CI)	*p* value	HR (95%CI)	*p* value	HR (95%CI)	*p* value
**NLR**						
Quartile1	1 (Reference)		1 (Reference)		1 (Reference)	
Quartile2	1.21 (1.08, 1.37)	0.0015	1.07 (0.95, 1.20)	0.2915	1.06 (0.94, 1.19)	0.3791
Quartile3	1.51 (1.35, 1.70)	<0.0001	1.22 (1.08, 1.37)	0.0010	1.22 (1.08, 1.37)	0.0010
Quartile4	2.34 (2.09, 2.61)	<0.0001	1.72 (1.53, 1.93)	<0.0001	1.73 (1.54, 1.94)	<0.0001
P for trend	1.38 (1.32, 1.43)	<0.0001	1.24 (1.19, 1.29)	<0.0001	1.24 (1.20, 1.29)	<0.0001
**MLR**						
Quartile1	1 (Reference)		1 (Reference)		1 (Reference)	
Quartile2	1.23 (1.09, 1.39)	0.0007	1.00 (0.88, 1.13)	0.9609	1.03 (0.91, 1.16)	0.6923
Quartile3	1.78 (1.58, 2.00)	<0.0001	1.27 (1.12, 1.43)	<0.0001	1.33 (1.17, 1.50)	<0.0001
Quartile4	2.76 (2.47, 3.09)	<0.0001	1.61 (1.43, 1.82)	<0.0001	1.71 (1.51, 1.93)	<0.0001
P for trend	24.01 (17.66, 32.65)	<0.0001	5.26 (3.77, 7.34)	<0.0001	6.10 (4.37, 8.53)	<0.0001
**PLR**						
Quartile1	1 (Reference)		1 (Reference)		1 (Reference)	
Quartile2	1.01 (0.90, 1.13)	0.9197	0.97 (0.87, 1.09)	0.6130	0.99 (0.89, 1.12)	0.9188
Quartile3	1.07 (0.96, 1.20)	0.2281	1.00 (0.89, 1.11)	0.9320	1.05 (0.94, 1.18)	0.3950
Quartile4	1.47 (1.32, 1.63)	<0.0001	1.23 (1.11, 1.37)	0.0002	1.28 (1.15, 1.43)	<0.0001
P for trend	1.00 (1.00, 1.00)	<0.0001	1.00 (1.00, 1.00)	<0.0001	1.00 (1.00, 1.00)	<0.0001
**SII**						
Quartile1	1 (Reference)		1 (Reference)		1 (Reference)	
Quartile2	1.17 (1.04, 1.31)	0.0078	1.10 (0.98, 1.24)	0.1041	1.10 (0.97, 1.23)	0.1266
Quartile3	1.22 (1.09, 1.37)	0.0005	1.17 (1.04, 1.31)	0.0085	1.16 (1.03, 1.30)	0.0119
Quartile4	1.64 (1.47, 1.83)	<0.0001	1.52 (1.36, 1.69)	<0.0001	1.50 (1.34, 1.68)	<0.0001
P for trend	1.00 (1.00, 1.00)	<0.0001	1.00 (1.00, 1.00)	<0.0001	1.00 (1.00, 1.00)	<0.0001
**SIRI**						
Quartile1	1 (Reference)		1 (Reference)		1 (Reference)	
Quartile2	1.43 (1.27, 1.61)	<0.0001	1.29 (1.15, 1.46)	<0.0001	1.25 (1.11, 1.41)	0.0002
Quartile3	1.61 (1.44, 1.81)	<0.0001	1.30 (1.15, 1.46)	<0.0001	1.28 (1.14, 1.45)	<0.0001
Quartile4	2.56 (2.29, 2.86)	<0.0001	1.92 (1.71, 2.16)	<0.0001	1.88 (1.67, 2.12)	<0.0001
P for trend	1.56 (1.48, 1.64)	<0.0001	1.36 (1.29, 1.43)	<0.0001	1.35 (1.28, 1.43)	<0.0001
**PIV**						
Quartile1	1 (Reference)		1 (Reference)		1 (Reference)	
Quartile2	1.14 (1.01, 1.28)	0.0301	1.11 (0.98, 1.24)	0.0908	1.10 (0.98, 1.23)	0.1139
Quartile3	1.30 (1.16, 1.46)	<0.0001	1.22 (1.08, 1.36)	0.0009	1.20 (1.07, 1.35)	0.0015
Quartile4	1.77 (1.59, 1.97)	<0.0001	1.58 (1.41, 1.76)	<0.0001	1.55 (1.38, 1.73)	<0.0001
P for trend	1.00 (1.00, 1.00)	<0.0001	1.00 (1.00, 1.00)	<0.0001	1.00 (1.00, 1.00)	<0.0001

Data are presented as HR (95% CI). Crude adjusts for none. Model 1 adjust for age, gender, and race. Model 2 adjust for Model 1 plus marital status, education level, family poverty income ratio, body mass index, smoking status, and alcohol status.

**Figure 1:**
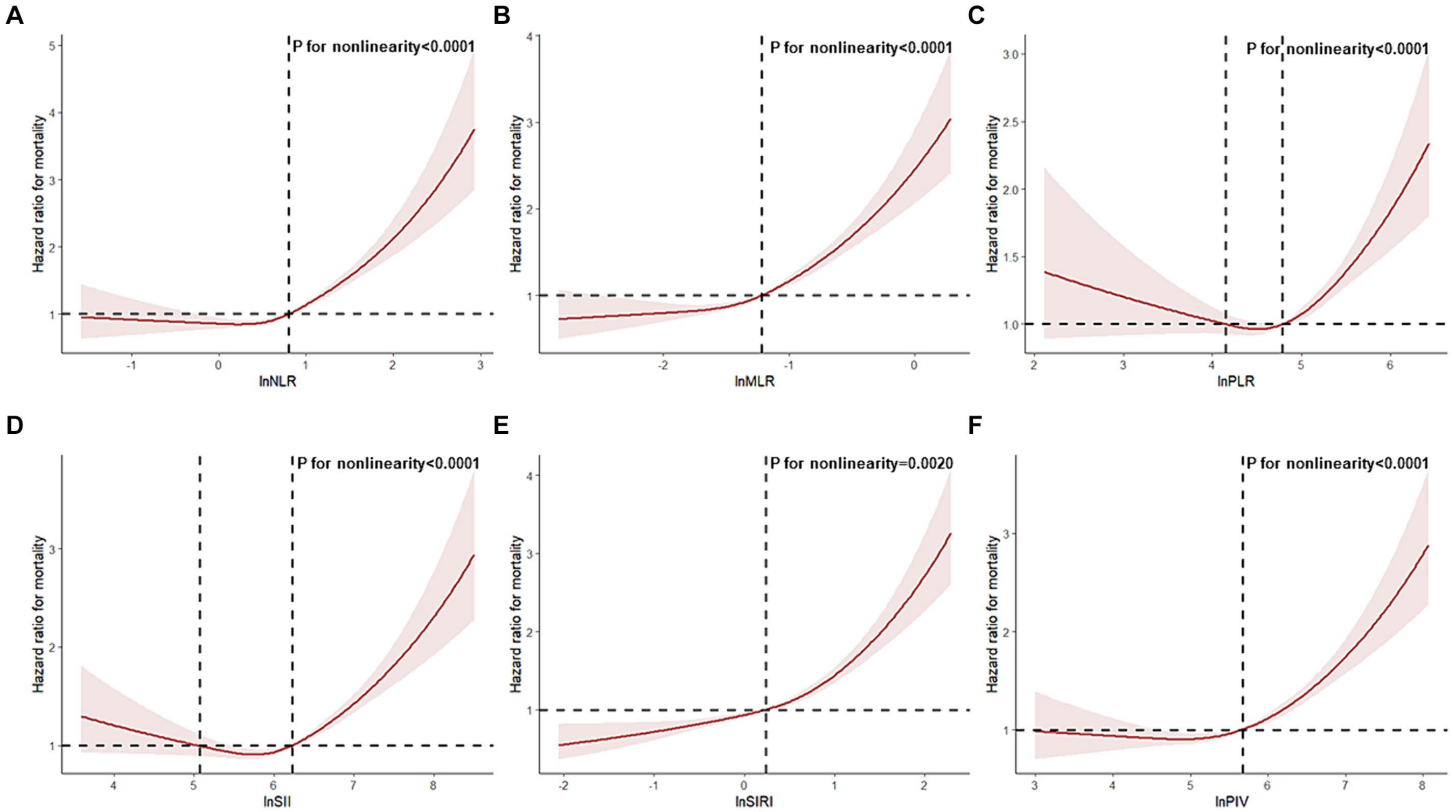
Restricted cubic spline analyses the association of complete blood cell count (CBC)-derived indicators (**A**: NLR; **B**: MLR; **C**: PLR; **D**: SII; **E**: SIRI; **F**: PIV) with all-cause mortality in middle-aged and older adult populations with frailty. Adjusted for adjusted for age, gender, race, marital status, education level, family poverty income ratio, body mass index, smoking status, and alcohol status.

CBC-derived inflammatory markers exhibited non-linear correlations with all-cause mortality in frail participants (P for non-linearity < 0.05, Figure 1). Additionally, we examined the relationship between CBC parameters and the risk of death in frail patients. We found that elevated neutrophil and monocyte counts were linked with an elevated risk of death in frail individuals, while increased lymphocyte and platelet counts were linked with a reduced risk of death in frail individuals (Supplementary Table S3).

### 3.4 Additional surveys of pre-frail participants

**Figure 2:**
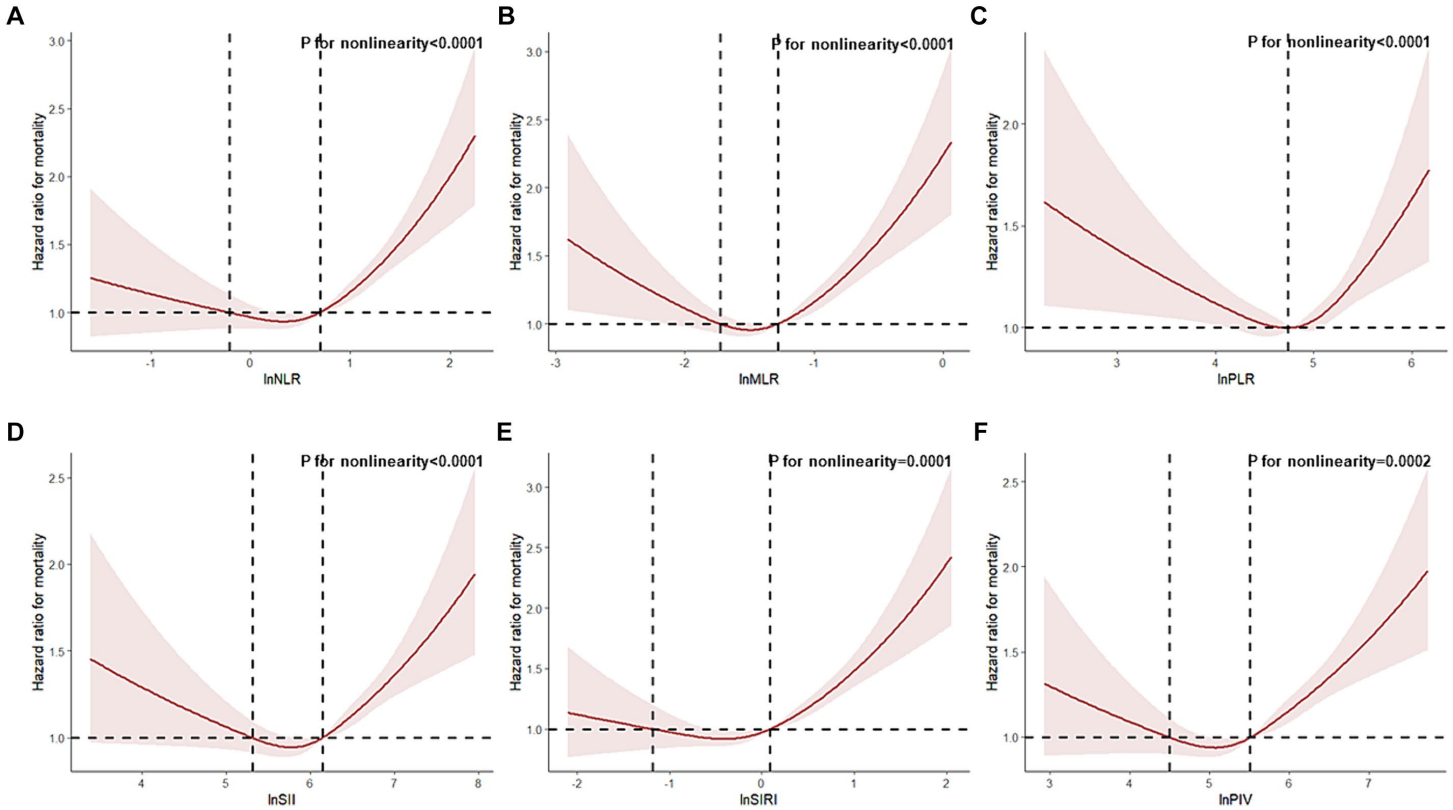
Restricted cubic spline analyses the association of complete blood cell count (CBC)-derived indicators (**A**: NLR; **B**: MLR; **C**: PLR; **D**: SII; **E**: SIRI; **F**: PIV) with all-cause mortality in middle-aged and older adult populations with pre-frailty. Adjusted for adjusted for age, gender, race, marital status, education level, family poverty income ratio, body mass index, smoking status, and alcohol status.

We conducted additional analyses involving 7,446 pre-frail participants (0.1 < FI < 0.25) to investigate the relationship between CBC parameters, their derived inflammatory markers, and all-cause mortality in pre-frail patients. In fully adjusted models, NLR, MLR, SII, SIRI, PIV, and neutrophil counts showed associations with an increased risk of all-cause mortality, while lymphocyte counts showed an association with a decreased risk of all-cause mortality. Their hazard ratios (HR) and 95% confidence intervals (95% CI) for death in the highest quartile compared with those in the lowest quartile of pre-frail participants were as follows: NLR (HR = 1.36, 95% CI: 1.19, 1.55), MLR (HR = 1.33, 95% CI: 1.16, 1.52), SII (HR = 1.24, 95% CI: 1.10, 1.41), SIRI (HR = 1.36, 95% CI: 1.18, 1.56), PIV (HR = 1.29, 95% CI: 1.13, 1.48), neutrophils (HR = 1.36, 95% CI: 1.19, 1.55), lymphocyte (HR = 0.80, 95% CI: 0.71, 0.91) (Table 4; Supplementary Table S4). A non-linear association was observed between all-cause mortality and CBC-derived inflammatory markers in pre-frail participants (P for non-linear <0.05, Figure 2).

**Table 4:** Associations of CBC-derived inflammatory markers with mortality in middle-aged and older adult populations with pre-frailty.

	Crude		Model 1		Model 2	
	HR (95%CI)	*p* value	HR (95%CI)	*p* value	HR (95%CI)	*p* value
**NLR**						
Quartile1	1 (Reference)		1 (Reference)		1 (Reference)	
Quartile2	1.02 (0.89, 1.17)	0.7895	0.96 (0.84, 1.11)	0.6099	0.97 (0.84, 1.12)	0.6582
Quartile3	1.31 (1.15, 1.49)	<0.0001	1.12 (0.98, 1.28)	0.0855	1.10 (0.96, 1.26)	0.1553
Quartile4	1.92 (1.70, 2.18)	<0.0001	1.39 (1.22, 1.59)	<0.0001	1.36 (1.19, 1.55)	<0.0001
P for trend	1.38 (1.31, 1.45)	<0.0001	1.19 (1.13, 1.25)	<0.0001	1.17 (1.11, 1.24)	<0.0001
**MLR**						
Quartile1	1 (Reference)		1 (Reference)		1 (Reference)	
Quartile2	1.17 (1.02, 1.34)	0.0291	0.98 (0.85, 1.13)	0.8080	1.01 (0.88, 1.16)	0.9051
Quartile3	1.49 (1.31, 1.70)	<0.0001	1.03 (0.90, 1.18)	0.6583	1.07 (0.93, 1.22)	0.3542
Quartile4	2.35 (2.08, 2.67)	<0.0001	1.29 (1.12, 1.47)	0.0003	1.33 (1.16, 1.52)	<0.0001
P for trend	28.11 (18.18, 43.47)	<0.0001	3.01 (1.87, 4.86)	<0.0001	3.27 (2.03, 5.27)	<0.0001
**PLR**						
Quartile1	1 (Reference)		1 (Reference)		1 (Reference)	
Quartile2	0.86 (0.75, 0.98)	0.0229	0.88 (0.77, 1.00)	0.0491	0.89 (0.78, 1.01)	0.0806
Quartile3	0.89 (0.78, 1.01)	0.0714	0.87 (0.76, 0.99)	0.0315	0.90 (0.79, 1.02)	0.0987
Quartile4	1.18 (1.05, 1.33)	0.0061	1.03 (0.91, 1.16)	0.6578	1.07 (0.95, 1.21)	0.2656
P for trend	1.00 (1.00, 1.00)	<0.0001	1.00 (1.00, 1.00)	0.2009	1.00 (1.00, 1.00)	0.0520
**SII**						
Quartile1	1 (Reference)		1 (Reference)		1 (Reference)	
Quartile2	0.90 (0.79, 1.04)	0.1471	0.96 (0.84, 1.11)	0.5939	0.94 (0.82, 1.07)	0.3548
Quartile3	1.11 (0.98, 1.26)	0.1083	1.08 (0.95, 1.24)	0.2322	1.04 (0.91, 1.18)	0.6028
Quartile4	1.47 (1.30, 1.66)	<0.0001	1.30 (1.15, 1.47)	<0.0001	1.24 (1.10, 1.41)	0.0007
P for trend	1.00 (1.00, 1.00)	<0.0001	1.00 (1.00, 1.00)	<0.0001	1.00 (1.00, 1.00)	<0.0001
**SIRI**						
Quartile1	1 (Reference)		1 (Reference)		1 (Reference)	
Quartile2	1.25 (1.09, 1.44)	0.0013	1.04 (0.90, 1.19)	0.6022	1.04 (0.90, 1.19)	0.6074
Quartile3	1.53 (1.34, 1.74)	<0.0001	1.19 (1.04, 1.37)	0.0138	1.16 (1.01, 1.34)	0.0323
Quartile4	2.25 (1.98, 2.55)	<0.0001	1.42 (1.24, 1.63)	<0.0001	1.36 (1.18, 1.56)	<0.0001
P for trend	1.62 (1.51, 1.74)	<0.0001	1.26 (1.17, 1.35)	<0.0001	1.22 (1.13, 1.31)	<0.0001
**PIV**						
Quartile1	1 (Reference)		1 (Reference)		1 (Reference)	
Quartile2	1.15 (1.00, 1.32)	0.0458	1.06 (0.92, 1.22)	0.4263	1.02 (0.89, 1.18)	0.7343
Quartile3	1.43 (1.25, 1.63)	<0.0001	1.20 (1.05, 1.38)	0.0083	1.16 (1.01, 1.33)	0.0372
Quartile4	1.83 (1.61, 2.08)	<0.0001	1.40 (1.22, 1.60)	<0.0001	1.29 (1.13, 1.48)	0.0002
P for trend	1.00 (1.00, 1.00)	<0.0001	1.00 (1.00, 1.00)	<0.0001	1.00 (1.00, 1.00)	<0.0001

Data are presented as HR (95% CI). Crude adjusts for none. Model 1 adjust for age, gender, and race. Model 2 adjust for Model 1 plus marital status, education level, family poverty income ratio, body mass index, smoking status, and alcohol status.

### 3.5 Prognostic value of CBC-derived inflammatory markers

**Figure 3:**
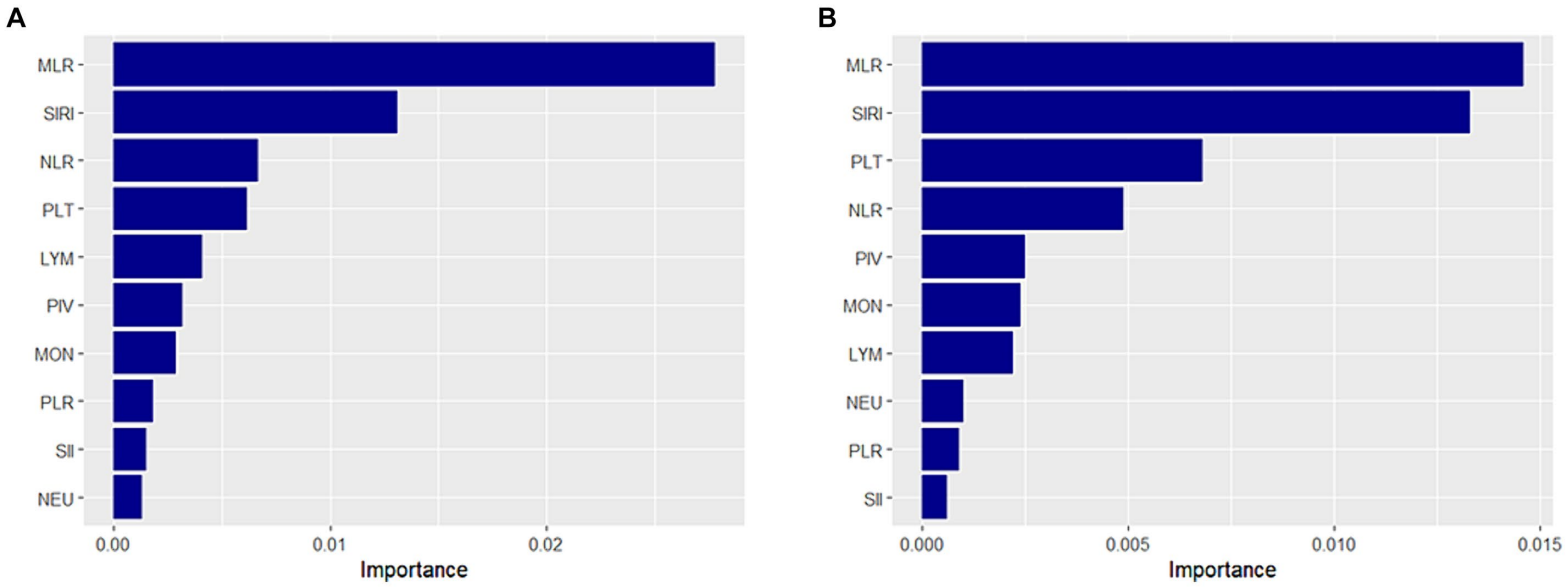
Prognostic value of complete blood cell count (CBC)-derived indicators. A random survival forest method was used to compare the value of CBC parameters and CBC-derived inflammatory markers in predicting all-cause mortality in middle-aged and older adult populations with frailty **(A)** and pre-frailty **(B)**.

We utilized the random survival forest (RSF) method to compare the predictive value of CBC parameters and their derived inflammatory markers for all-cause mortality in the frail middle-aged and older adult population. Our findings indicate that the monocyte-to-lymphocyte ratio (MLR) exhibits the highest prognostic value for predicting all-cause mortality in both frailty and pre-frailty populations (Figure 3). Therefore, MLR may serve as a valuable clinical indicator for predicting the risk of death in frail populations.

### 3.6 Sensitivity analyses

After we adjusted the FI threshold and redefined frailty, the association of CBC parameters and their derived inflammatory markers with the risk of frailty and death remained unchanged (Supplementary Tables S5, S6). A new FI was constructed after excluding platelet counts, and reclassify the frail population. We observed similar results in the adjusted full model (Supplementary Tables S7, S8).

## 4 Discussion

CBC-derived inflammatory markers integrate multiple parameters, which have the advantage of their ability to capture the complex interactions between different cell types in the blood, which is more informative than any single measurement. Therefore, in this study of a nationally representative population in the United States, we focused on the relationship of CBC-derived inflammatory markers to the risk of frailty and mortality in middle-aged and older adults. We found that elevated levels of NLR, MLR, PLR, SII, SIRI, and PIV were positively associated with frailty risk, whereas lower levels of PLR were negatively associated with frailty risk. Among frail patients, higher levels of NLR, MLR, PLR, SII, SIRI, and PIV were found to be associated with an increased risk of death. Additionally, the results of our survival prediction modeling analyses indicated that MLR was the most predictive inflammatory marker for mortality risk in this population. These findings suggest that CBC-derived inflammatory markers, especially MLR, may serve as useful clinical indicators for assessing mortality risk in frail patients. In conclusion, our study demonstrates the potential utility of CBC-derived inflammatory markers as indicators for assessing both frailty and mortality risk.

The development of frailty is linked to various physiological alterations, including chronic inflammation and age-related changes (22). Previously, several studies have demonstrated that levels of inflammatory markers such as IL-6, CRP (C-reactive protein), and TNF-α are markedly elevated in older and frail individuals (23–25). Recently, several studies have investigated the association between CBC-derived inflammatory markers and frailty, finding significant associations with NLR, PLR, SII, and PIV (15, 26, 27). However, most of these studies had small sample sizes and focused on individuals with specific diseases and hospitalizations. This study targeted a broad population to comprehensively assess the association of multiple CBC-derived inflammatory markers with frailty risk and prognosis.

In our investigation, we observed a u-shaped association between PLR and SII levels and mortality in frail patients. This suggests that elevated and reduced levels of PLR and SII may both increase the risk of death. However, in the non-linear association between NLR, MLR, SIRI, and PIV and mortality, an increased risk of death was observed only at high levels. These results suggest that excessive immune-inflammatory responses in frail populations pose significant health risks. Research has indicated that excessive inflammation promotes chronic inflammation and may serve as a potential etiologic factor of sarcopenia associated with frailty (28, 29). Chronic inflammation contributes to cardiovascular risk events and is a major driver of cancer-related frailty (30, 31). Considering the risk of an inflammatory state, maintaining appropriate levels of CBC-derived inflammatory markers is crucial for reducing mortality in frail patients.

In our additional analysis focused on pre-frail patients, we found that elevated levels of NLR, MLR, SII, SIRI, PIV, and neutrophil counts were associated with an elevated risk of all-cause mortality, while increased levels of lymphocyte counts were associated with a reduced risk of all-cause mortality. We observed a u-shaped association between all six CBC-derived inflammatory markers and the risk of all-cause mortality in pre-frail participants. In contrast to the frail population, pre-frail participants with excessively low levels of NLR, MLR, SIRI, and PIV may also face an increased risk of mortality. Notably, excessive levels of CBC-derived inflammatory markers may have a more profound effect on increased mortality in frail patients compared to pre-frail patients.

Among the four CBC parameters and six CBC-derived markers of inflammation, MLR stands out as the most valuable predictor of the risk of death in both frailty and pre-frail individuals. MLR is the ratio of monocytes to lymphocytes; elevated MLR indicates either an increase in monocytes or a decrease in lymphocytes. Research has shown that monocytes and macrophages mediate molecular inflammatory pathways in frail patients (32, 33). Additionally, frailty is associated with elevated monocyte counts (34). Frailty is associated with the aging of multiple physiological structures and functions, particularly immune senescence (35). Changes in lymphocyte numbers and function characterize immune senescence and contribute to the development of frailty (36). A study found a negative correlation between lymphocyte counts and frailty and that lymphocyte counts were lower in frail older adults (37). This is likely because the thymus undergoes atrophy due to aging, resulting in reduced lymphocyte production and functional changes (38). Thus, elevated MLR reflects an increased inflammatory response and the progression of frailty, indicating an elevated risk of death in frail patients (39). These findings suggest that MLR may be associated with the underlying biological mechanisms of frailty. Therefore, MLR is valuable for predicting the risk of death in frail patients.

Our research has several strengths. First, our study population is derived from a large, nationally representative health survey, resulting in findings that are more reliable and representative. Second, CBC-derived inflammatory markers comprise multiple parameters, potentially offering a more comprehensive assessment of immune and inflammatory responses than a single indicator. Third, we employed a restricted cubic spline to investigate the nonlinear relationship between CBC-derived inflammatory markers and mortality risk. Fourth, we conducted several sensitivity analyses to mitigate bias resulting from constructing FI and determining thresholds. Finally, we examined the association of multiple CBC parameters and their derived inflammatory markers with the risk of frailty and mortality. Using the RSF method, we identified MLR as the most valuable predictor of all-cause mortality in the frail middle-aged and older adult population.

However, this study also has several limitations. First, the construction of the frailty index lacks uniformity, and the threshold values for frailty are arbitrarily determined, potentially biasing the selection of frail populations. Second, although this study adjusted for multiple confounders, potential confounding factors may still exist. Third, the use of one-time CBC parameters to calculate CBC-derived inflammatory markers may lead to bias. Fourth, we failed to account for differences in the nonlinear relationship between CBC-derived inflammatory markers and the risk of death in frail and pre-frail populations.

## 5 Conclusion

The findings of this study indicate that elevated levels of six CBC-derived inflammatory markers are correlated with increased all-cause mortality in middle and old-aged individuals with frailty. Elevated levels of MLR, NLR, SIRI, SII, and PIV were linked to increased all-cause mortality in middle-aged and older adult individuals in a pre-frail state. MLR aids in identifying high-risk individuals in frail populations and is a cost-effective and readily accessible marker. Our study offers further evidence supporting the potential utility of CBC-derived inflammatory markers in predicting frailty prognosis.

## Data availability statement

Publicly available datasets were analyzed in this study. This data can be found here: https://www.cdc.gov/nchs/nhanes.

## Ethics statement

The studies involving humans were approved by NCHS Research Ethics Committee. The studies were conducted in accordance with the local legislation and institutional requirements. The participants provided their written informed consent to participate in this study.

## Author contributions

YT: Writing – original draft, Writing – review & editing. YZ: Writing – original draft, Writing – review & editing. WS: Writing – review & editing. TZ: Writing – review & editing. ZX: Writing – review & editing. LJ: Writing – review & editing. LL: Writing – review & editing. DL: Writing – review & editing. QW: Writing – review & editing.

## References

[1] Hoogendijk EO Afilalo J Ensrud KE Kowal P Onder G Fried LP. Frailty: implications for clinical practice and public health. Lancet (North American ed). (2019) 394:1365–75. doi: 10.1016/S0140-6736(19)31786-6 31609228

[2] Song X Mitnitski A Rockwood K. Prevalence and 10-year outcomes of frailty in older adults in relation to deficit accumulation. J Am Geriatr Soc. (2010) 58:681–7. doi: 10.1111/j.1532-5415.2010.02764.x, PMID: 20345864

[3] Clegg A Young J Iliffe S Rikkert MO Rockwood K. Frailty in elderly people. Lancet Lond Engl. (2013) 381:752–62. doi: 10.1016/S0140-6736(12)62167-9, PMID: 23395245 PMC4098658

[4] Bergman H Ferrucci L Guralnik J Hogan DB Hummel S Karunananthan S. Frailty: an emerging research and clinical paradigm--issues and controversies. J Gerontol A Biol Sci Med Sci. (2007) 62:731–7. doi: 10.1093/gerona/62.7.731, PMID: 17634320 PMC2645660

[5] Dent E Martin FC Bergman H Woo J Romero-Ortuno R Walston JD. Management of frailty: opportunities, challenges, and future directions. Lancet (North American ed). (2019) 394:1376–86. doi: 10.1016/S0140-6736(19)31785-4, PMID: 31609229

[6] Fried LP Tangen CM Walston J Newman AB Hirsch C Gottdiener J. Frailty in older adults: evidence for a phenotype. J Gerontol A Biol Sci Med Sci. (2001) 56:M146–57. doi: 10.1093/gerona/56.3.m146 11253156

[7] Samson LD Boots AMH Verschuren WMM Picavet HSJ Engelfriet P Buisman A-M. Frailty is associated with elevated CRP trajectories and higher numbers of neutrophils and monocytes. Exp Gerontol. (2019) 125:110674. doi: 10.1016/j.exger.2019.110674, PMID: 31336145

[8] Welstead M Muniz-Terrera G Russ TC Corley J Taylor AM Gale CR. Inflammation as a risk factor for the development of frailty in the Lothian birth cohort 1936. Exp Gerontol. (2020) 139:111055. doi: 10.1016/j.exger.2020.111055, PMID: 32795628 PMC7456784

[9] Bektas A Schurman SH Sen R Ferrucci L. Aging, inflammation and the environment. Exp Gerontol. (2018) 105:10–8. doi: 10.1016/j.exger.2017.12.015, PMID: 29275161 PMC5909704

[10] Soysal P Arik F Smith L Jackson SE Isik AT. Inflammation, frailty and cardiovascular disease. Adv Exp Med Biol. (2020) 1216:55–64. doi: 10.1007/978-3-030-33330-0_7 31894547

[11] Ferrucci L Fabbri E. Inflammageing: chronic inflammation in ageing, cardiovascular disease, and frailty. Nat Rev Cardiol. (2018) 15:505–22. doi: 10.1038/s41569-018-0064-2, PMID: 30065258 PMC6146930

[12] Ke J Qiu F Fan W Wei S. Associations of complete blood cell count-derived inflammatory biomarkers with asthma and mortality in adults: a population-based study. Front Immunol. (2023) 14:1205687. doi: 10.3389/fimmu.2023.1205687, PMID: 37575251 PMC10416440

[13] Li Q Ma X Shao Q Yang Z Wang Y Gao F. Prognostic impact of multiple lymphocyte-based inflammatory indices in acute coronary syndrome patients. Front Cardiovasc Med. (2022) 9:811790. doi: 10.3389/fcvm.2022.811790, PMID: 35592392 PMC9110784

[14] Asik Z Özen M. Evaluation of frailty and neutrophil-to-lymphocyte and platelet-to-lymphocyte ratios relationship in elderly people. Nagoya J Med Sci. (2022) 84:101–10. doi: 10.18999/nagjms.84.1.101, PMID: 35392007 PMC8971038

[15] Okyar Baş A Güner M Ceylan S Hafızoğlu M Şahiner Z Doğu BB. Pan-immune inflammation value; a novel biomarker reflecting inflammation associated with frailty. Aging Clin Exp Res. (2023) 35:1641–9. doi: 10.1007/s40520-023-02457-0 37289361

[16] Zheng Z Luo H Xue Q. U-shaped association of systemic immune-inflammation index levels with cancer-related and all-cause mortality in middle-aged and older individuals with frailty. Arch Gerontol Geriatr. (2024) 116:105228. doi: 10.1016/j.archger.2023.105228 39491075

[17] Jayanama K Theou O Godin J Mayo A Cahill L Rockwood K. Relationship of body mass index with frailty and all-cause mortality among middle-aged and older adults. BMC Med. (2022) 20:404. doi: 10.1186/s12916-022-02596-7, PMID: 36280863 PMC9594976

[18] Blodgett JM Theou O Howlett SE Rockwood K. A frailty index from common clinical and laboratory tests predicts increased risk of death across the life course. GeroScience. (2017) 39:447–55. doi: 10.1007/s11357-017-9993-7, PMID: 28866737 PMC5636769

[19] Fan J Yu C Guo Y Bian Z Sun Z Yang L. Frailty index and all-cause and cause-specific mortality in Chinese adults: a prospective cohort study. Lancet Public Health. (2020) 5:e650–60. doi: 10.1016/S2468-2667(20)30113-4, PMID: 33271078 PMC7708389

[20] Shi SM Olivieri-Mui B McCarthy EP Kim DH. Changes in a frailty index and association with mortality. J Am Geriatr Soc. (2021) 69:1057–62. doi: 10.1111/jgs.17002, PMID: 33377190 PMC8071066

[21] Cheng W Bu X Xu C Wen G Kong F Pan H. Higher systemic immune-inflammation index and systemic inflammation response index levels are associated with stroke prevalence in the asthmatic population: a cross-sectional analysis of the NHANES 1999-2018. Front Immunol. (2023) 14:1191130. doi: 10.3389/fimmu.2023.1191130, PMID: 37600830 PMC10436559

[22] Taylor JA Greenhaff PL Bartlett DB Jackson TA Duggal NA Lord JM. Multisystem physiological perspective of human frailty and its modulation by physical activity. Physiol Rev. (2023) 103:1137–91. doi: 10.1152/physrev.00037.2021, PMID: 36239451 PMC9886361

[23] Picca A Coelho-Junior HJ Calvani R Marzetti E Vetrano DL. Biomarkers shared by frailty and sarcopenia in older adults: a systematic review and meta-analysis. Ageing Res Rev. (2022) 73:101530. doi: 10.1016/j.arr.2021.101530, PMID: 34839041

[24] Soysal P Stubbs B Lucato P Luchini C Solmi M Peluso R. Inflammation and frailty in the elderly: a systematic review and meta-analysis. Ageing Res Rev. (2016) 31:1–8. doi: 10.1016/j.arr.2016.08.006, PMID: 27592340

[25] Xu Y Wang M Chen D Jiang X Xiong Z. Inflammatory biomarkers in older adults with frailty: a systematic review and meta-analysis of cross-sectional studies. Aging Clin Exp Res. (2022) 34:971–87. doi: 10.1007/s40520-021-02022-7, PMID: 34981430

[26] Guan L Liu Q Yao Y Wang L Peng Y Chen S. Do neutrophil to lymphocyte ratio and platelet to lymphocyte ratio associate with frailty in elderly inpatient with comorbidity? Exp Gerontol. (2022) 169:111955. doi: 10.1016/j.exger.2022.111955, PMID: 36122594

[27] Zhang H Hao M Hu Z Li Y Jiang X Wang J. Association of immunity markers with the risk of incident frailty: the Rugao longitudinal aging study. Immun Ageing. (2022) 19:1. doi: 10.1186/s12979-021-00257-6, PMID: 34980175 PMC8722120

[28] Wilson D Jackson T Sapey E Lord JM. Frailty and sarcopenia: the potential role of an aged immune system. Ageing Res Rev. (2017) 36:1–10. doi: 10.1016/j.arr.2017.01.006, PMID: 28223244

[29] Byrne T Cooke J Bambrick P McNeela E Harrison M. Circulating inflammatory biomarker responses in intervention trials in frail and sarcopenic older adults: a systematic review and meta-analysis. Exp Gerontol. (2023) 177:112199. doi: 10.1016/j.exger.2023.112199, PMID: 37156445

[30] Cushman M Arnold AM Psaty BM Manolio TA Kuller LH Burke GL. C-reactive protein and the 10-year incidence of coronary heart disease in older men and women: the cardiovascular health study. Circulation. (2005) 112:25–31. doi: 10.1161/CIRCULATIONAHA.104.504159, PMID: 15983251

[31] Di Meglio A Vaz-Luis I. Systemic inflammation and cancer-related frailty: shifting the paradigm toward precision survivorship medicine. ESMO Open. (2024) 9:102205. doi: 10.1016/j.esmoop.2023.102205, PMID: 38194879 PMC10820355

[32] Qu T Yang H Walston JD Fedarko NS Leng SX. Upregulated monocytic expression of CXC chemokine ligand 10 (CXCL-10) and its relationship with serum interleukin-6 levels in the syndrome of frailty. Cytokine. (2009) 46:319–24. doi: 10.1016/j.cyto.2009.02.015, PMID: 19342252 PMC3159181

[33] Qu T Walston JD Yang H Fedarko NS Xue Q-L Beamer BA. Upregulated *ex vivo* expression of stress-responsive inflammatory pathway genes by LPS-challenged CD14(+) monocytes in frail older adults. Mech Ageing Dev. (2009) 130:161–6. doi: 10.1016/j.mad.2008.10.005, PMID: 19027777 PMC2673566

[34] Leng SX Xue Q-L Tian J Huang Y Yeh S-H Fried LP. Associations of neutrophil and monocyte counts with frailty in community-dwelling disabled older women: results from the Women’s health and aging studies I. Exp Gerontol. (2009) 44:511–6. doi: 10.1016/j.exger.2009.05.005, PMID: 19457449

[35] Tran Van Hoi E De Glas NA Portielje JEA Van Heemst D Van Den Bos F Jochems SP. Biomarkers of the ageing immune system and their association with frailty - a systematic review. Exp Gerontol. (2023) 176:112163. doi: 10.1016/j.exger.2023.112163, PMID: 37028607

[36] Núñez J Sastre C D’Ascoli G Ruiz V Bonanad C Miñana G. Relation of low lymphocyte count to frailty and its usefulness as a prognostic biomarker in patients >65 years of age with acute coronary syndrome. Am J Cardiol. (2020) 125:1033–8. doi: 10.1016/j.amjcard.2020.01.006, PMID: 31959430

[37] Navarro-Martínez R Serrano-Carrascosa M Buigues C Fernández-Garrido J Sánchez-Martínez V Castelló-Domenech AB. Frailty syndrome is associated with changes in peripheral inflammatory markers in prostate cancer patients undergoing androgen deprivation therapy. Urol Oncol. (2019) 37:976–87. doi: 10.1016/j.urolonc.2019.08.005, PMID: 31521528

[38] Vallejo AN. Immune remodeling: lessons from repertoire alterations during chronological aging and in immune-mediated disease. Trends Mol Med. (2007) 13:94–102. doi: 10.1016/j.molmed.2007.01.005, PMID: 17267287

[39] Bingol O Ozdemir G Kulakoglu B Keskin OH Korkmaz I Kilic E. Admission neutrophil-to-lymphocyte ratio and monocyte-to-lymphocyte ratio to predict 30-day and 1-year mortality in geriatric hip fractures. Injury. (2020) 51:2663–7. doi: 10.1016/j.injury.2020.07.048, PMID: 32739153

