# Supplementary material for: Association between complete blood count-derived inflammatory markers and the risk of frailty and mortality in middle-aged and older adults
Source: Front Public Health. 2024 Jul 31;12:1427546. doi: 10.3389/fpubh.2024.1427546 (PMC11323558; doi:10.3389/fpubh.2024.1427546)
Supplement: Supplementary file 1 [file Data_Sheet_1.docx]

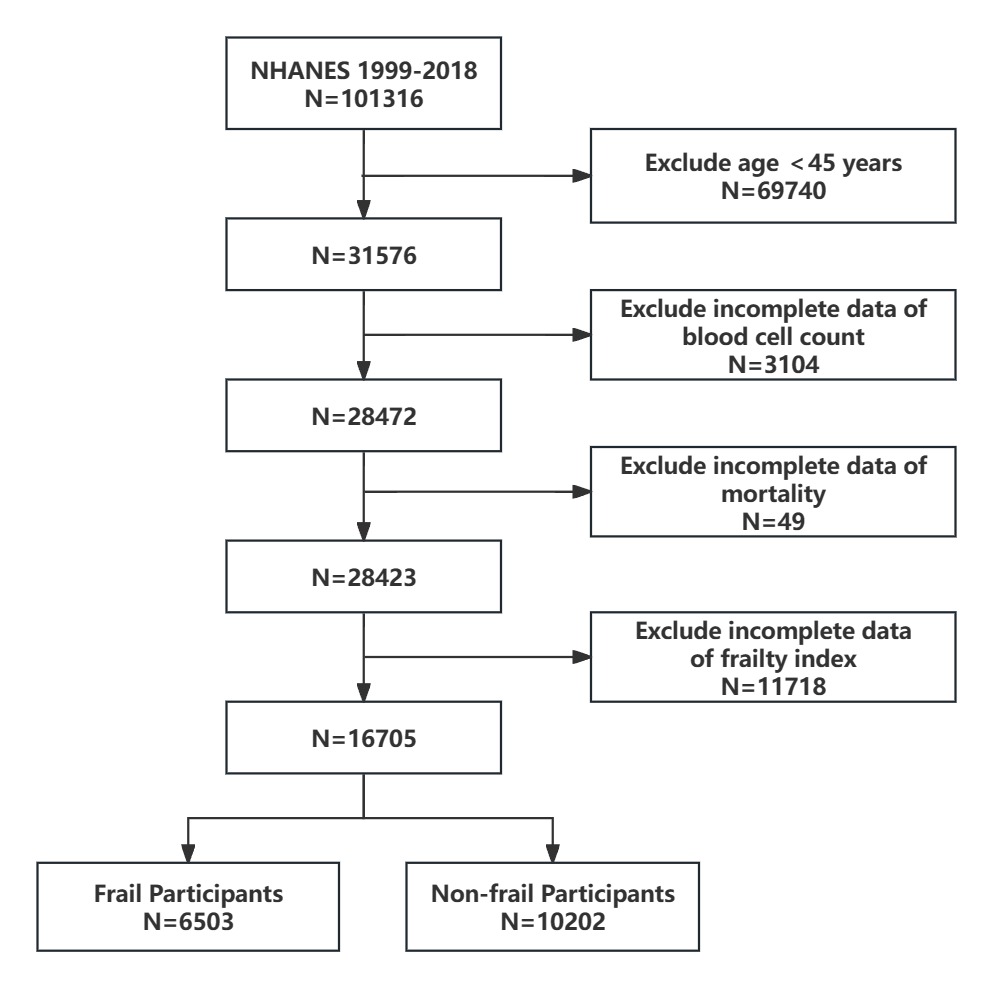


**Supplementary Figure S1.** Flowchart of the study participants

**Supplementary Table S1.** Variables in the 36-item frailty index and their respective scorings

| Variable | Scoring |
| --- | --- |
| Self-reported Frailty Index items |  |
| 1. Angina/angina pectoris | Yes=1, No=0 |
| 2. Heart attack | Yes=1, No=0 |
| 3. Coronary heart disease | Yes=1, No=0 |
| 4. Stroke | Yes=1, No=0 |
| 5. Thyroid condition | Yes=1, No=0 |
| 6. Cancer | Yes=1, No=0 |
| 7. Arthritis | Yes=1, No=0 |
| 8. High blood pressure | Yes=1, No=0 |
| 9. Diabetes mellitus | Yes=1, No=0 |
| 10. Weak/failing kidneys | Yes=1, No=0 |
| 11. Confusion or inability to remember things | Yes=1, No=0 |
| 12. Difficulty managing money | Yes=1, No=0 |
| 13. Difficulty stooping, crouching, kneeling | Yes=1, No=0 |
| 14. Difficulty lifting or carrying | Yes=1, No=0 |
| 15. Difficulty walking between rooms on same floor | Yes=1, No=0 |
| 16. Difficulty standing up from an armless chair | Yes=1, No=0 |
| 17. Difficulty getting in and out of bed | Yes=1, No=0 |
| 18. Difficulty dressing yourself | Yes=1, No=0 |
| 19. Difficulty grasping/holding small objects | Yes=1, No=0 |
| 20. Difficulty attending social events | Yes=1, No=0 |
| 21. Self-reported health | Fair, poor=1, Excellent, Very good, good=0 |
| 22. Frequency of healthcare use | None=0, 1-2=0.5, 3 and more=1 |
| 23. Health compared to 1 year ago | Worse=1, About the same, better=0 |
| 24. Overnight hospital stays | Yes=1, No=0 |
| 25. Medications | None=0, 1-3=0.5, 4 and more=1 |
| Laboratory Frailty Index items |  |
| 26. Pulse rate | 60-99 bpm=0， Other=1 |
| 27. Systolic blood pressure | 90-140 mmHg=0，Other=1 |
| 28. Pulse pressure | 30-60 mmHg=0， Other=1 |
| 29. Platelet count SI | 150-450 unit 1000 cells/uL=0， Other=1 |
| 30. Blood urea nitrogen | 3-20 mg/dL=0， Other=1 |
| 31. Bicarbonate | ≤28 mmol/L=0，Other=1 |
| 32. Red cell distribution width | ≤14.6%=0，Other=1 |
| 33. Lactate dehydrogenase | ≤190 U/L=0，Other=1 |
| 34. Alkaline phosphatase | ≤115 U/L=0，Other=1 |
| 35. Uric acid | M: 240-510 umol/L =0，Other=1, |
|  | F: 160-430 umol/L=0， Other=1 |
| 36. Total calcium | 2.0-2.5 mmol/L=0， Other=1 |
|  |  |

F = female; M = male

**Table S2.** Associations between CBC parameters and frailty risk in middle-aged and elderly populations

|  | Crude |  | Model 1 |  | Model 2 |  |
| --- | --- | --- | --- | --- | --- | --- |
|  | OR (95%CI) | P value | OR (95%CI) | P value | OR (95%CI) | P value |
| Neutrophils |  |  |  |  |  |  |
| Quartile1 | 1(Reference) |  | 1(Reference) |  | 1(Reference) |  |
| Quartile2 | 1.08 (0.98, 1.19) | 0.1021 | 1.14 (1.04, 1.26) | 0.0055 | 1.07 (0.97, 1.18) | 0.1955 |
| Quartile3 | 1.39 (1.27, 1.53) | <0.0001 | 1.52 (1.38, 1.67) | <0.0001 | 1.30 (1.18, 1.43) | <0.0001 |
| Quartile4 | 1.95 (1.78, 2.14) | <0.0001 | 2.21 (2.01, 2.43) | <0.0001 | 1.74 (1.57, 1.92) | <0.0001 |
| P for trend | 1.22 (1.19, 1.25) | <0.0001 | 1.27 (1.23, 1.30) | <0.0001 | 1.18 (1.15, 1.21) | <0.0001 |
| Monocyte |  |  |  |  |  |  |
| Quartile1 | 1(Reference) |  | 1(Reference) |  | 1(Reference) |  |
| Quartile2 | 0.95 (0.84, 1.06) | 0.3516 | 0.97 (0.86, 1.09) | 0.6369 | 0.92 (0.82, 1.05) | 0.2165 |
| Quartile3 | 1.13 (1.00, 1.28) | 0.0569 | 1.20 (1.05, 1.36) | 0.006 | 1.06 (0.93, 1.22) | 0.3749 |
| Quartile4 | 1.43 (1.27, 1.61) | <0.0001 | 1.56 (1.38, 1.76) | <0.0001 | 1.31 (1.15, 1.49) | <0.0001 |
| P for trend | 2.82 (2.30, 3.45) | <0.0001 | 3.37 (2.73, 4.16) | <0.0001 | 2.31 (1.85, 2.88) | <0.0001 |
| Lymphocyte |  |  |  |  |  |  |
| Quartile1 | 1(Reference) |  | 1(Reference) |  | 1(Reference) |  |
| Quartile2 | 0.78 (0.71, 0.86) | <0.0001 | 0.79 (0.72, 0.87) | <0.0001 | 0.75 (0.68, 0.82) | <0.0001 |
| Quartile3 | 0.70 (0.64, 0.77) | <0.0001 | 0.71 (0.65, 0.78) | <0.0001 | 0.62 (0.57, 0.69) | <0.0001 |
| Quartile4 | 0.90 (0.83, 0.99) | 0.0245 | 0.92 (0.84, 1.01) | 0.068 | 0.72 (0.66, 0.80) | <0.0001 |
| P for trend | 0.96 (0.91, 1.01) | 0.1417 | 0.97 (0.92, 1.03) | 0.3218 | 0.83 (0.79, 0.89) | <0.0001 |
| Platelet |  |  |  |  |  |  |
| Quartile1 | 1(Reference) |  | 1(Reference) |  | 1(Reference) |  |
| Quartile2 | 0.72 (0.66, 0.78) | <0.0001 | 0.70 (0.64, 0.77) | <0.0001 | 0.70 (0.64, 0.77) | <0.0001 |
| Quartile3 | 0.78 (0.72, 0.85) | <0.0001 | 0.76 (0.69, 0.83) | <0.0001 | 0.74 (0.67, 0.81) | <0.0001 |
| Quartile4 | 0.89 (0.82, 0.98) | 0.0119 | 0.86 (0.79, 0.95) | 0.0016 | 0.83 (0.75, 0.91) | <0.0001 |
| P for trend | 1.00 (1.00, 1.00) | 0.1874 | 1.00 (1.00, 1.00) | 0.0525 | 1.00 (1.00, 1.00) | 0.0038 |
|  |  |  |  |  |  |  |

Data are presented as OR (95% CI). Crude adjusts for none. Model 1: adjusted for age, gender, and race; Model 2 was adjusted for model 1 variables plus marital status, education level, family poverty income ratio, body mass index, smoking status, and alcohol status.

**Table S3.** Associations of CBC parameters with all-cause mortality in middle-aged and elderly populations with frailty.

|  | Crude |  | Model 1 |  | Model 2 |  |
| --- | --- | --- | --- | --- | --- | --- |
|  | HR (95%CI) | P value | HR (95%CI) | P value | HR (95%CI) | P value |
| Neutrophils |  |  |  |  |  |  |
| Quartile1 | 1(Reference) |  | 1(Reference) |  | 1(Reference) |  |
| Quartile2 | 1.14 (1.02, 1.27) | 0.0221 | 1.02 (0.91, 1.14) | 0.6920 | 1.00 (0.89, 1.12) | 0.9482 |
| Quartile3 | 1.20 (1.07, 1.34) | 0.0015 | 1.13 (1.00, 1.26) | 0.0430 | 1.08 (0.96, 1.21) | 0.2069 |
| Quartile4 | 1.38 (1.23, 1.53) | <0.0001 | 1.49 (1.34, 1.67) | <0.0001 | 1.40 (1.25, 1.57) | <0.0001 |
| P for trend | 1.09 (1.06, 1.12) | <0.0001 | 1.12 (1.09, 1.16) | <0.0001 | 1.11 (1.07, 1.14) | <0.0001 |
| Monocyte |  |  |  |  |  |  |
| Quartile1 | 1(Reference) |  | 1(Reference) |  | 1(Reference) |  |
| Quartile2 | 1.15 (1.03, 1.29) | 0.0158 | 1.08 (0.96, 1.21) | 0.1972 | 1.09 (0.97, 1.23) | 0.1461 |
| Quartile3 | 1.20 (1.07, 1.35) | 0.0021 | 1.09 (0.97, 1.22) | 0.1691 | 1.08 (0.96, 1.22) | 0.1752 |
| Quartile4 | 1.46 (1.31, 1.61) | <0.0001 | 1.27 (1.15, 1.41) | <0.0001 | 1.24 (1.12, 1.38) | <0.0001 |
| P for trend | 2.45 (1.94, 3.10) | <0.0001 | 1.80 (1.42, 2.29) | <0.0001 | 1.68 (1.32, 2.14) | <0.0001 |
| Lymphocyte |  |  |  |  |  |  |
| Quartile1 | 1(Reference) |  | 1(Reference) |  | 1(Reference) |  |
| Quartile2 | 0.69 (0.63, 0.76) | <0.0001 | 0.81 (0.74, 0.90) | <0.0001 | 0.79 (0.71, 0.88) | <0.0001 |
| Quartile3 | 0.54 (0.48, 0.60) | <0.0001 | 0.72 (0.65, 0.81) | <0.0001 | 0.71 (0.63, 0.79) | <0.0001 |
| Quartile4 | 0.47 (0.43, 0.53) | <0.0001 | 0.74 (0.66, 0.82) | <0.0001 | 0.68 (0.61, 0.76) | <0.0001 |
| P for trend | 0.62 (0.58, 0.66) | <0.0001 | 0.82 (0.77, 0.88) | <0.0001 | 0.79 (0.73, 0.84) | <0.0001 |
| Platelet |  |  |  |  |  |  |
| Quartile1 | 1(Reference) |  | 1(Reference) |  | 1(Reference) |  |
| Quartile2 | 0.77 (0.69, 0.85) | <0.0001 | 0.84 (0.76, 0.94) | 0.0019 | 0.86 (0.78, 0.96) | 0.0069 |
| Quartile3 | 0.68 (0.61, 0.75) | <0.0001 | 0.85 (0.77, 0.95) | 0.0039 | 0.83 (0.74, 0.92) | 0.0006 |
| Quartile4 | 0.62 (0.56, 0.69) | <0.0001 | 0.90 (0.80, 1.00) | 0.0525 | 0.88 (0.79, 0.99) | 0.0308 |
| P for trend | 1.00 (1.00, 1.00) | <0.0001 | 1.00 (1.00, 1.00) | 0.1039 | 1.00 (1.00, 1.00) | 0.0368 |
|  |  |  |  |  |  |  |

Data are presented as HR (95% CI). Crude adjusts for none. Model 1: adjusted for age, gender, and race; Model 2 was adjusted for model 1 variables plus marital status, education level, family poverty income ratio, body mass index, smoking status, and alcohol status.

**Table S4.** Associations of CBC parameters with all-cause mortality in middle-aged and elderly populations with pre-frailty.

|  | Crude |  | Model 1 |  | Model 2 |  |
| --- | --- | --- | --- | --- | --- | --- |
|  | HR (95%CI) | P value | HR (95%CI) | P value | HR (95%CI) | P value |
| Neutrophils |  |  |  |  |  |  |
| Quartile1 | 1(Reference) |  | 1(Reference) |  | 1(Reference) |  |
| Quartile2 | 1.13 (0.99, 1.30) | 0.0681 | 1.08 (0.94, 1.24) | 0.2674 | 1.07 (0.94, 1.23) | 0.3045 |
| Quartile3 | 1.35 (1.19, 1.53) | <0.0001 | 1.23 (1.08, 1.41) | 0.0015 | 1.19 (1.04, 1.35) | 0.0106 |
| Quartile4 | 1.53 (1.35, 1.73) | <0.0001 | 1.49 (1.31, 1.69) | <0.0001 | 1.36 (1.19, 1.55) | <0.0001 |
| P for trend | 1.15 (1.11, 1.19) | <0.0001 | 1.14 (1.10, 1.19) | <0.0001 | 1.11 (1.06, 1.15) | <0.0001 |
| Monocyte |  |  |  |  |  |  |
| Quartile1 | 1(Reference) |  | 1(Reference) |  | 1(Reference) |  |
| Quartile2 | 0.89 (0.73, 1.08) | 0.2268 | 0.83 (0.68, 1.00) | 0.0503 | 0.86 (0.71, 1.04) | 0.1253 |
| Quartile3 | 1.12 (0.95, 1.32) | 0.1909 | 0.90 (0.76, 1.07) | 0.2360 | 0.93 (0.78, 1.10) | 0.3723 |
| Quartile4 | 1.63 (1.37, 1.93) | <0.0001 | 1.08 (0.91, 1.29) | 0.3888 | 1.04 (0.88, 1.25) | 0.6243 |
| P for trend | 3.51 (2.73, 4.51) | <0.0001 | 1.62 (1.24, 2.10) | 0.0003 | 1.39 (1.07, 1.80) | 0.0151 |
| Lymphocyte |  |  |  |  |  |  |
| Quartile1 | 1(Reference) |  | 1(Reference) |  | 1(Reference) |  |
| Quartile2 | 0.74 (0.66, 0.83) | <0.0001 | 0.85 (0.76, 0.96) | 0.0097 | 0.82 (0.73, 0.93) | 0.0016 |
| Quartile3 | 0.64 (0.57, 0.72) | <0.0001 | 0.87 (0.77, 0.99) | 0.0309 | 0.84 (0.74, 0.95) | 0.0048 |
| Quartile4 | 0.58 (0.51, 0.65) | <0.0001 | 0.89 (0.78, 1.01) | 0.0623 | 0.80 (0.71, 0.91) | 0.0008 |
| P for trend | 0.71 (0.66, 0.77) | <0.0001 | 0.94 (0.87, 1.02) | 0.1146 | 0.88 (0.82, 0.96) | 0.0024 |
| Platelet |  |  |  |  |  |  |
| Quartile1 | 1(Reference) |  | 1(Reference) |  | 1(Reference) |  |
| Quartile2 | 0.82 (0.73, 0.93) | 0.0020 | 0.93 (0.83, 1.06) | 0.2857 | 0.94 (0.83, 1.07) | 0.3463 |
| Quartile3 | 0.69 (0.61, 0.79) | <0.0001 | 0.86 (0.75, 0.97) | 0.0166 | 0.84 (0.74, 0.95) | 0.0067 |
| Quartile4 | 0.69 (0.61, 0.78) | <0.0001 | 0.97 (0.86, 1.10) | 0.6438 | 0.92 (0.81, 1.04) | 0.1738 |
| P for trend | 1.00 (1.00, 1.00) | <0.0001 | 1.00 (1.00, 1.00) | 0.6131 | 1.00 (1.00, 1.00) | 0.1295 |
|  |  |  |  |  |  |  |

Data are presented as HR (95% CI). Crude adjusts for none. Model 1: adjusted for age, gender, and race; Model 2 was adjusted for model 1 variables plus marital status, education level, family poverty income ratio, body mass index, smoking status, and alcohol status.

**Table S5.** Associations of CBC parameters and CBC-derived inflammatory markers with frailty risk in middle-aged and elderly populations.

|  | Crude |  | Model 1 |  | Model 2 |  |
| --- | --- | --- | --- | --- | --- | --- |
|  | OR (95%CI) | P value | OR (95%CI) | P value | OR (95%CI) | P value |
| NLR |  |  |  |  |  |  |
| Quartile1 | 1(Reference) |  | 1(Reference) |  | 1(Reference) |  |
| Quartile2 | 1.02 (0.93, 1.11) | 0.7334 | 1.07 (0.98, 1.17) | 0.1224 | 1.07 (0.97, 1.17) | 0.1638 |
| Quartile3 | 1.17 (1.08, 1.28) | 0.0003 | 1.27 (1.16, 1.39) | <0.0001 | 1.23 (1.12, 1.35) | <0.0001 |
| Quartile4 | 1.80 (1.65, 1.96) | <0.0001 | 1.98 (1.81, 2.18) | <0.0001 | 1.92 (1.74, 2.11) | <0.0001 |
| P for trend | 1.30 (1.25, 1.35) | <0.0001 | 1.35 (1.30, 1.40) | <0.0001 | 1.33 (1.28, 1.38) | <0.0001 |
| MLR |  |  |  |  |  |  |
| Quartile1 | 1(Reference) |  | 1(Reference) |  | 1(Reference) |  |
| Quartile2 | 0.92 (0.85, 1.01) | 0.0678 | 0.96 (0.88, 1.05) | 0.3929 | 1.02 (0.93, 1.12) | 0.6782 |
| Quartile3 | 1.07 (0.98, 1.17) | 0.1103 | 1.15 (1.05, 1.25) | 0.0034 | 1.21 (1.10, 1.33) | <0.0001 |
| Quartile4 | 1.52 (1.39, 1.65) | <0.0001 | 1.65 (1.50, 1.82) | <0.0001 | 1.82 (1.64, 2.01) | <0.0001 |
| P for trend | 5.44 (4.03, 7.35) | <0.0001 | 7.46 (5.37, 10.37) | <0.0001 | 10.08 (7.14, 14.25) | <0.0001 |
| PLR |  |  |  |  |  |  |
| Quartile1 | 1(Reference) |  | 1(Reference) |  | 1(Reference) |  |
| Quartile2 | 0.82 (0.75, 0.89) | <0.0001 | 0.80 (0.74, 0.88) | <0.0001 | 0.85 (0.77, 0.93) | 0.0004 |
| Quartile3 | 0.82 (0.75, 0.89) | <0.0001 | 0.80 (0.74, 0.88) | <0.0001 | 0.91 (0.83, 0.99) | 0.0340 |
| Quartile4 | 0.98 (0.90, 1.07) | 0.6381 | 0.93 (0.85, 1.01) | 0.0836 | 1.10 (1.00, 1.20) | 0.0452 |
| P for trend | 1.00 (1.00, 1.00) | 0.6602 | 1.00 (1.00, 1.00) | 0.4209 | 1.00 (1.00, 1.00) | 0.0022 |
| SII |  |  |  |  |  |  |
| Quartile1 | 1(Reference) |  | 1(Reference) |  | 1(Reference) |  |
| Quartile2 | 0.94 (0.86, 1.03) | 0.1696 | 0.97 (0.89, 1.06) | 0.5029 | 0.94 (0.85, 1.03) | 0.1602 |
| Quartile3 | 1.09 (1.00, 1.18) | 0.0605 | 1.12 (1.02, 1.22) | 0.0124 | 1.06 (0.97, 1.17) | 0.1869 |
| Quartile4 | 1.56 (1.43, 1.70) | <0.0001 | 1.62 (1.49, 1.78) | <0.0001 | 1.52 (1.38, 1.67) | <0.0001 |
| P for trend | 1.00 (1.00, 1.00) | <0.0001 | 1.00 (1.00, 1.00) | <0.0001 | 1.00 (1.00, 1.00) | <0.0001 |
| SIRI |  |  |  |  |  |  |
| Quartile1 | 1(Reference) |  | 1(Reference) |  | 1(Reference) |  |
| Quartile2 | 1.01 (0.92, 1.10) | 0.9019 | 1.08 (0.99, 1.18) | 0.0856 | 1.02 (0.92, 1.12) | 0.7463 |
| Quartile3 | 1.31 (1.21, 1.43) | <0.0001 | 1.48 (1.35, 1.62) | <0.0001 | 1.34 (1.22, 1.47) | <0.0001 |
| Quartile4 | 1.94 (1.78, 2.12) | <0.0001 | 2.25 (2.05, 2.48) | <0.0001 | 1.96 (1.78, 2.16) | <0.0001 |
| P for trend | 1.51 (1.44, 1.59) | <0.0001 | 1.64 (1.56, 1.73) | <0.0001 | 1.52 (1.44, 1.61) | <0.0001 |
| PIV |  |  |  |  |  |  |
| Quartile1 | 1(Reference) |  | 1(Reference) |  | 1(Reference) |  |
| Quartile2 | 1.04 (0.95, 1.13) | 0.3877 | 1.08 (0.99, 1.18) | 0.0939 | 1.02 (0.93, 1.12) | 0.6538 |
| Quartile3 | 1.23 (1.13, 1.34) | <0.0001 | 1.30 (1.19, 1.42) | <0.0001 | 1.18 (1.08, 1.30) | 0.0004 |
| Quartile4 | 1.74 (1.60, 1.90) | <0.0001 | 1.89 (1.72, 2.07) | <0.0001 | 1.65 (1.50, 1.82) | <0.0001 |
| P for trend | 1.00 (1.00, 1.00) | <0.0001 | 1.00 (1.00, 1.00) | <0.0001 | 1.00 (1.00, 1.00) | <0.0001 |
| Neutrophils |  |  |  |  |  |  |
| Quartile1 | 1(Reference) |  | 1(Reference) |  | 1(Reference) |  |
| Quartile2 | 1.08 (0.99, 1.18) | 0.0987 | 1.13 (1.03, 1.24) | 0.0079 | 1.05 (0.96, 1.16) | 0.2937 |
| Quartile3 | 1.34 (1.23, 1.47) | <0.0001 | 1.45 (1.33, 1.59) | <0.0001 | 1.23 (1.12, 1.35) | <0.0001 |
| Quartile4 | 1.89 (1.73, 2.07) | <0.0001 | 2.13 (1.94, 2.34) | <0.0001 | 1.66 (1.50, 1.83) | <0.0001 |
| P for trend | 1.21 (1.18, 1.24) | <0.0001 | 1.25 (1.22, 1.28) | <0.0001 | 1.16 (1.13, 1.19) | <0.0001 |
| Monocyte |  |  |  |  |  |  |
| Quartile1 | 1(Reference) |  | 1(Reference) |  | 1(Reference) |  |
| Quartile2 | 0.93 (0.83, 1.05) | 0.2366 | 0.95 (0.85, 1.07) | 0.3922 | 0.90 (0.80, 1.01) | 0.0839 |
| Quartile3 | 1.15 (1.02, 1.30) | 0.0257 | 1.20 (1.06, 1.37) | 0.0036 | 1.07 (0.93, 1.22) | 0.3460 |
| Quartile4 | 1.42 (1.27, 1.60) | <0.0001 | 1.53 (1.35, 1.72) | <0.0001 | 1.27 (1.12, 1.45) | 0.0002 |
| P for trend | 2.81 (2.31, 3.43) | <0.0001 | 3.30 (2.69, 4.06) | <0.0001 | 2.23 (1.80, 2.77) | <0.0001 |
| Lymphocyte |  |  |  |  |  |  |
| Quartile1 | 1(Reference) |  | 1(Reference) |  | 1(Reference) |  |
| Quartile2 | 0.77 (0.70, 0.84) | <0.0001 | 0.78 (0.71, 0.85) | <0.0001 | 0.74 (0.67, 0.81) | <0.0001 |
| Quartile3 | 0.69 (0.63, 0.75) | <0.0001 | 0.71 (0.65, 0.77) | <0.0001 | 0.62 (0.56, 0.68) | <0.0001 |
| Quartile4 | 0.89 (0.81, 0.97) | 0.0066 | 0.92 (0.84, 1.00) | 0.0546 | 0.72 (0.65, 0.79) | <0.0001 |
| P for trend | 0.95 (0.90, 1.00) | 0.0643 | 0.97 (0.92, 1.03) | 0.3241 | 0.83 (0.79, 0.88) | <0.0001 |
| Platelet |  |  |  |  |  |  |
| Quartile1 | 1(Reference) |  | 1(Reference) |  | 1(Reference) |  |
| Quartile2 | 0.73 (0.67, 0.79) | <0.0001 | 0.71 (0.65, 0.78) | <0.0001 | 0.71 (0.65, 0.78) | <0.0001 |
| Quartile3 | 0.77 (0.71, 0.84) | <0.0001 | 0.74 (0.68, 0.81) | <0.0001 | 0.72 (0.66, 0.79) | <0.0001 |
| Quartile4 | 0.88 (0.80, 0.95) | 0.0023 | 0.85 (0.77, 0.93) | 0.0003 | 0.81 (0.73, 0.89) | <0.0001 |
| P for trend | 1.00 (1.00, 1.00) | 0.0492 | 1.00 (1.00, 1.00) | 0.0105 | 1.00 (1.00, 1.00) | 0.0003 |
|  |  |  |  |  |  |  |

After redefining the FI cut-off value of frailty, the association of CBC parameters and CBC-derived inflammatory markers with the risk of frailty in middle-aged and elderly people was analyzed. Data are presented as OR (95% CI). Crude adjusts for none. Model 1: adjusted for age, gender, and race; Model 2 was adjusted for model 1 variables plus marital status, education level, family poverty income ratio, body mass index, smoking status, and alcohol status.

**Table S6.** Associations of CBC parameters and CBC-derived inflammatory markers with all-cause mortality in middle-aged and elderly populations with frailty.

|  | Crude |  | Model 1 |  | Model 2 |  |
| --- | --- | --- | --- | --- | --- | --- |
|  | HR (95%CI) | P value | HR (95%CI) | P value | HR (95%CI) | P value |
| NLR |  |  |  |  |  |  |
| Quartile1 | 1(Reference) |  | 1(Reference) |  | 1(Reference) |  |
| Quartile2 | 1.22 (1.09, 1.37) | 0.0004 | 1.09 (0.97, 1.21) | 0.1545 | 1.08 (0.96, 1.21) | 0.1926 |
| Quartile3 | 1.52 (1.36, 1.69) | <0.0001 | 1.21 (1.09, 1.36) | 0.0006 | 1.21 (1.09, 1.35) | 0.0006 |
| Quartile4 | 2.38 (2.14, 2.63) | <0.0001 | 1.72 (1.54, 1.91) | <0.0001 | 1.72 (1.54, 1.91) | <0.0001 |
| P for trend | 1.39 (1.34, 1.44) | <0.0001 | 1.24 (1.19, 1.29) | <0.0001 | 1.24 (1.19, 1.29) | <0.0001 |
| MLR |  |  |  |  |  |  |
| Quartile1 | 1(Reference) |  | 1(Reference) |  | 1(Reference) |  |
| Quartile2 | 1.21 (1.08, 1.35) | 0.0013 | 0.98 (0.87, 1.10) | 0.7278 | 1.02 (0.91, 1.14) | 0.7911 |
| Quartile3 | 1.70 (1.53, 1.90) | <0.0001 | 1.21 (1.09, 1.36) | 0.0006 | 1.27 (1.13, 1.41) | <0.0001 |
| Quartile4 | 2.76 (2.49, 3.06) | <0.0001 | 1.58 (1.42, 1.77) | <0.0001 | 1.68 (1.50, 1.88) | <0.0001 |
| P for trend | 25.03 (18.79, 33.35) | <0.0001 | 5.08 (3.72, 6.93) | <0.0001 | 5.87 (4.30, 8.02) | <0.0001 |
| PLR |  |  |  |  |  |  |
| Quartile1 | 1(Reference) |  | 1(Reference) |  | 1(Reference) |  |
| Quartile2 | 1.02 (0.91, 1.13) | 0.7753 | 0.97 (0.87, 1.08) | 0.5658 | 0.99 (0.89, 1.11) | 0.9001 |
| Quartile3 | 1.04 (0.94, 1.16) | 0.4276 | 0.97 (0.87, 1.08) | 0.5899 | 1.03 (0.92, 1.14) | 0.6368 |
| Quartile4 | 1.45 (1.32, 1.60) | <0.0001 | 1.21 (1.10, 1.34) | 0.0002 | 1.27 (1.15, 1.40) | <0.0001 |
| P for trend | 1.00 (1.00, 1.00) | <0.0001 | 1.00 (1.00, 1.00) | <0.0001 | 1.00 (1.00, 1.00) | <0.0001 |
| SII |  |  |  |  |  |  |
| Quartile1 | 1(Reference) |  | 1(Reference) |  | 1(Reference) |  |
| Quartile2 | 1.15 (1.03, 1.28) | 0.0098 | 1.13 (1.01, 1.26) | 0.0268 | 1.13 (1.01, 1.25) | 0.0316 |
| Quartile3 | 1.24 (1.11, 1.37) | <0.0001 | 1.18 (1.06, 1.32) | 0.0019 | 1.16 (1.05, 1.30) | 0.0052 |
| Quartile4 | 1.64 (1.48, 1.82) | <0.0001 | 1.52 (1.37, 1.68) | <0.0001 | 1.49 (1.34, 1.65) | <0.0001 |
| P for trend | 1.00 (1.00, 1.00) | <0.0001 | 1.00 (1.00, 1.00) | <0.0001 | 1.00 (1.00, 1.00) | <0.0001 |
| SIRI |  |  |  |  |  |  |
| Quartile1 | 1(Reference) |  | 1(Reference) |  | 1(Reference) |  |
| Quartile2 | 1.38 (1.23, 1.54) | <0.0001 | 1.22 (1.09, 1.36) | 0.0005 | 1.19 (1.07, 1.33) | 0.0021 |
| Quartile3 | 1.62 (1.45, 1.80) | <0.0001 | 1.29 (1.16, 1.44) | <0.0001 | 1.28 (1.14, 1.42) | <0.0001 |
| Quartile4 | 2.55 (2.30, 2.83) | <0.0001 | 1.88 (1.68, 2.09) | <0.0001 | 1.83 (1.64, 2.04) | <0.0001 |
| P for trend | 1.57 (1.50, 1.65) | <0.0001 | 1.36 (1.30, 1.43) | <0.0001 | 1.35 (1.29, 1.42) | <0.0001 |
| PIV |  |  |  |  |  |  |
| Quartile1 | 1(Reference) |  | 1(Reference) |  | 1(Reference) |  |
| Quartile2 | 1.15 (1.03, 1.28) | 0.0109 | 1.10 (0.98, 1.22) | 0.0932 | 1.09 (0.97, 1.21) | 0.1348 |
| Quartile3 | 1.31 (1.18, 1.45) | <0.0001 | 1.21 (1.09, 1.35) | 0.0005 | 1.19 (1.07, 1.32) | 0.0015 |
| Quartile4 | 1.81 (1.64, 2.00) | <0.0001 | 1.60 (1.44, 1.78) | <0.0001 | 1.56 (1.40, 1.73) | <0.0001 |
| P for trend | 1.00 (1.00, 1.00) | <0.0001 | 1.00 (1.00, 1.00) | <0.0001 | 1.00 (1.00, 1.00) | <0.0001 |
| Neutrophils |  |  |  |  |  |  |
| Quartile1 | 1(Reference) |  | 1(Reference) |  | 1(Reference) |  |
| Quartile2 | 1.18 (1.06, 1.30) | 0.0023 | 1.04 (0.94, 1.16) | 0.4171 | 1.01 (0.91, 1.12) | 0.8303 |
| Quartile3 | 1.24 (1.12, 1.37) | <0.0001 | 1.16 (1.05, 1.29) | 0.0041 | 1.11 (1.00, 1.23) | 0.0450 |
| Quartile4 | 1.44 (1.30, 1.59) | <0.0001 | 1.56 (1.41, 1.73) | <0.0001 | 1.44 (1.29, 1.60) | <0.0001 |
| P for trend | 1.10 (1.07, 1.13) | <0.0001 | 1.13 (1.10, 1.16) | <0.0001 | 1.11 (1.08, 1.14) | <0.0001 |
| Monocyte |  |  |  |  |  |  |
| Quartile1 | 1(Reference) |  | 1(Reference) |  | 1(Reference) |  |
| Quartile2 | 1.16 (1.04, 1.29) | 0.0080 | 1.06 (0.96, 1.19) | 0.2546 | 1.08 (0.97, 1.20) | 0.1697 |
| Quartile3 | 1.20 (1.08, 1.34) | 0.0007 | 1.07 (0.96, 1.19) | 0.2141 | 1.07 (0.96, 1.20) | 0.2028 |
| Quartile4 | 1.51 (1.37, 1.66) | <0.0001 | 1.30 (1.18, 1.43) | <0.0001 | 1.26 (1.14, 1.39) | <0.0001 |
| P for trend | 2.69 (2.16, 3.35) | <0.0001 | 1.93 (1.54, 2.41) | <0.0001 | 1.77 (1.41, 2.21) | <0.0001 |
| Lymphocyte |  |  |  |  |  |  |
| Quartile1 | 1(Reference) |  | 1(Reference) |  | 1(Reference) |  |
| Quartile2 | 0.70 (0.64, 0.77) | <0.0001 | 0.83 (0.76, 0.91) | 0.0001 | 0.80 (0.73, 0.88) | <0.0001 |
| Quartile3 | 0.55 (0.50, 0.60) | <0.0001 | 0.74 (0.67, 0.82) | <0.0001 | 0.72 (0.65, 0.80) | <0.0001 |
| Quartile4 | 0.49 (0.44, 0.53) | <0.0001 | 0.77 (0.70, 0.85) | <0.0001 | 0.71 (0.64, 0.78) | <0.0001 |
| P for trend | 0.63 (0.59, 0.67) | <0.0001 | 0.84 (0.79, 0.90) | <0.0001 | 0.80 (0.75, 0.86) | <0.0001 |
| Platelet |  |  |  |  |  |  |
| Quartile1 | 1(Reference) |  | 1(Reference) |  | 1(Reference) |  |
| Quartile2 | 0.78 (0.71, 0.86) | <0.0001 | 0.86 (0.78, 0.95) | 0.0027 | 0.87 (0.79, 0.97) | 0.0082 |
| Quartile3 | 0.68 (0.61, 0.75) | <0.0001 | 0.85 (0.77, 0.94) | 0.0012 | 0.82 (0.74, 0.90) | <0.0001 |
| Quartile4 | 0.64 (0.58, 0.70) | <0.0001 | 0.94 (0.84, 1.04) | 0.2019 | 0.92 (0.83, 1.02) | 0.0938 |
| P for trend | 1.00 (1.00, 1.00) | <0.0001 | 1.00 (1.00, 1.00) | 0.2910 | 1.00 (1.00, 1.00) | 0.0880 |
|  |  |  |  |  |  |  |

After redefining the FI cut-off value of frailty, the associations of CBC parameters and CBC-derived inflammatory markers with all-cause mortality in middle-aged and elderly populations with frailty was analyzed. Data are presented as HR (95% CI). Crude adjusts for none. Model 1: adjusted for age, gender, and race; Model 2 was adjusted for model 1 variables plus marital status, education level, family poverty income ratio, body mass index, smoking status, and alcohol status.

**Table S7.** Associations of CBC parameters and CBC-derived inflammatory markers with frailty risk in middle-aged and elderly populations.

|  | Crude |  | Model 1 |  | Model 2 |  |
| --- | --- | --- | --- | --- | --- | --- |
|  | OR (95%CI) | P value | OR (95%CI) | P value | OR (95%CI) | P value |
| NLR |  |  |  |  |  |  |
| Quartile1 | 1(Reference) |  | 1(Reference) |  | 1(Reference) |  |
| Quartile2 | 1.01 (0.93, 1.11) | 0.7765 | 1.08 (0.99, 1.19) | 0.0995 | 1.08 (0.98, 1.19) | 0.1279 |
| Quartile3 | 1.18 (1.08, 1.29) | 0.0002 | 1.30 (1.19, 1.43) | <0.0001 | 1.26 (1.14, 1.39) | <0.0001 |
| Quartile4 | 1.80 (1.65, 1.97) | <0.0001 | 2.03 (1.85, 2.23) | <0.0001 | 1.98 (1.79, 2.18) | <0.0001 |
| P for trend | 1.30 (1.26, 1.35) | <0.0001 | 1.37 (1.32, 1.42) | <0.0001 | 1.35 (1.30, 1.40) | <0.0001 |
| MLR |  |  |  |  |  |  |
| Quartile1 | 1(Reference) |  | 1(Reference) |  | 1(Reference) |  |
| Quartile2 | 0.93 (0.85, 1.02) | 0.1368 | 0.98 (0.90, 1.08) | 0.7450 | 1.05 (0.95, 1.15) | 0.3412 |
| Quartile3 | 1.07 (0.98, 1.17) | 0.1364 | 1.17 (1.06, 1.28) | 0.0013 | 1.23 (1.12, 1.36) | <0.0001 |
| Quartile4 | 1.47 (1.35, 1.61) | <0.0001 | 1.66 (1.51, 1.83) | <0.0001 | 1.83 (1.66, 2.03) | <0.0001 |
| P for trend | 4.89 (3.60, 6.64) | <0.0001 | 7.47 (5.34, 10.44) | <0.0001 | 10.18 (7.16, 14.48) | <0.0001 |
| PLR |  |  |  |  |  |  |
| Quartile1 | 1(Reference) |  | 1(Reference) |  | 1(Reference) |  |
| Quartile2 | 0.86 (0.79, 0.94) | 0.001 | 0.85 (0.78, 0.93) | 0.0004 | 0.90 (0.82, 0.99) | 0.0293 |
| Quartile3 | 0.86 (0.79, 0.94) | 0.0011 | 0.85 (0.78, 0.93) | 0.0005 | 0.97 (0.88, 1.06) | 0.4999 |
| Quartile4 | 1.04 (0.95, 1.14) | 0.3772 | 1.00 (0.91, 1.09) | 0.9935 | 1.20 (1.09, 1.32) | 0.0002 |
| P for trend | 1.00 (1.00, 1.00) | 0.1027 | 1.00 (1.00, 1.00) | 0.4479 | 1.00 (1.00, 1.00) | <0.0001 |
| SII |  |  |  |  |  |  |
| Quartile1 | 1(Reference) |  | 1(Reference) |  | 1(Reference) |  |
| Quartile2 | 0.99 (0.90, 1.08) | 0.8447 | 1.03 (0.94, 1.13) | 0.5632 | 1.00 (0.91, 1.10) | 0.9610 |
| Quartile3 | 1.16 (1.06, 1.27) | 0.0009 | 1.22 (1.11, 1.33) | <0.0001 | 1.17 (1.06, 1.28) | 0.0017 |
| Quartile4 | 1.66 (1.52, 1.81) | <0.0001 | 1.76 (1.61, 1.93) | <0.0001 | 1.66 (1.51, 1.83) | <0.0001 |
| P for trend | 1.00 (1.00, 1.00) | <0.0001 | 1.00 (1.00, 1.00) | <0.0001 | 1.00 (1.00, 1.00) | <0.0001 |
| SIRI |  |  |  |  |  |  |
| Quartile1 | 1(Reference) |  | 1(Reference) |  | 1(Reference) |  |
| Quartile2 | 1.05 (0.96, 1.15) | 0.3143 | 1.15 (1.04, 1.26) | 0.0042 | 1.08 (0.98, 1.19) | 0.1124 |
| Quartile3 | 1.44 (1.32, 1.57) | <0.0001 | 1.66 (1.51, 1.82) | <0.0001 | 1.52 (1.38, 1.68) | <0.0001 |
| Quartile4 | 1.98 (1.81, 2.16) | <0.0001 | 2.38 (2.16, 2.62) | <0.0001 | 2.09 (1.89, 2.31) | <0.0001 |
| P for trend | 1.52 (1.45, 1.60) | <0.0001 | 1.67 (1.59, 1.76) | <0.0001 | 1.56 (1.48, 1.65) | <0.0001 |
| PIV |  |  |  |  |  |  |
| Quartile1 | 1(Reference) |  | 1(Reference) |  | 1(Reference) |  |
| Quartile2 | 1.09 (1.00, 1.19) | 0.0634 | 1.15 (1.05, 1.26) | 0.0036 | 1.09 (0.99, 1.20) | 0.0742 |
| Quartile3 | 1.37 (1.25, 1.50) | <0.0001 | 1.48 (1.35, 1.62) | <0.0001 | 1.36 (1.24, 1.50) | <0.0001 |
| Quartile4 | 1.86 (1.70, 2.04) | <0.0001 | 2.07 (1.89, 2.27) | <0.0001 | 1.84 (1.67, 2.02) | <0.0001 |
| P for trend | 1.00 (1.00, 1.00) | <0.0001 | 1.00 (1.00, 1.00) | <0.0001 | 1.00 (1.00, 1.00) | <0.0001 |
| Neutrophils |  |  |  |  |  |  |
| Quartile1 | 1(Reference) |  | 1(Reference) |  | 1(Reference) |  |
| Quartile2 | 1.11 (1.01, 1.22) | 0.0318 | 1.18 (1.07, 1.29) | 0.0008 | 1.10 (1.00, 1.22) | 0.0577 |
| Quartile3 | 1.42 (1.29, 1.55) | <0.0001 | 1.55 (1.41, 1.71) | <0.0001 | 1.33 (1.20, 1.46) | <0.0001 |
| Quartile4 | 2.02 (1.85, 2.21) | <0.0001 | 2.30 (2.09, 2.53) | <0.0001 | 1.81 (1.64, 2.00) | <0.0001 |
| P for trend | 1.23 (1.20, 1.26) | <0.0001 | 1.28 (1.25, 1.31) | <0.0001 | 1.19 (1.16, 1.23) | <0.0001 |
| Monocyte |  |  |  |  |  |  |
| Quartile1 | 1(Reference) |  | 1(Reference) |  | 1(Reference) |  |
| Quartile2 | 0.96 (0.86, 1.08) | 0.5445 | 0.99 (0.88, 1.12) | 0.9273 | 0.95 (0.84, 1.07) | 0.4031 |
| Quartile3 | 1.16 (1.02, 1.32) | 0.0228 | 1.24 (1.09, 1.41) | 0.0013 | 1.10 (0.96, 1.26) | 0.1714 |
| Quartile4 | 1.47 (1.30, 1.65) | <0.0001 | 1.61 (1.42, 1.82) | <0.0001 | 1.36 (1.19, 1.55) | <0.0001 |
| P for trend | 2.92 (2.38, 3.58) | <0.0001 | 3.55 (2.88, 4.38) | <0.0001 | 2.44 (1.95, 3.04) | <0.0001 |
| Lymphocyte |  |  |  |  |  |  |
| Quartile1 | 1(Reference) |  | 1(Reference) |  | 1(Reference) |  |
| Quartile2 | 0.78 (0.71, 0.86) | <0.0001 | 0.79 (0.72, 0.86) | <0.0001 | 0.74 (0.67, 0.82) | <0.0001 |
| Quartile3 | 0.71 (0.65, 0.78) | <0.0001 | 0.72 (0.66, 0.79) | <0.0001 | 0.63 (0.57, 0.70) | <0.0001 |
| Quartile4 | 0.92 (0.84, 1.00) | 0.0552 | 0.93 (0.85, 1.01) | 0.1005 | 0.73 (0.66, 0.80) | <0.0001 |
| P for trend | 0.97 (0.92, 1.02) | 0.2836 | 0.98 (0.93, 1.03) | 0.4559 | 0.84 (0.79, 0.89) | <0.0001 |
| Platelet |  |  |  |  |  |  |
| Quartile1 | 1(Reference) |  | 1(Reference) |  | 1(Reference) |  |
| Quartile2 | 0.78 (0.71, 0.85) | <0.0001 | 0.77 (0.70, 0.84) | <0.0001 | 0.77 (0.70, 0.85) | <0.0001 |
| Quartile3 | 0.85 (0.78, 0.93) | 0.0004 | 0.82 (0.75, 0.90) | <0.0001 | 0.81 (0.74, 0.89) | <0.0001 |
| Quartile4 | 0.97 (0.89, 1.06) | 0.4642 | 0.93 (0.85, 1.02) | 0.1426 | 0.90 (0.82, 0.99) | 0.0306 |
| P for trend | 1.00 (1.00, 1.00) | 0.7718 | 1.00 (1.00, 1.00) | 0.6582 | 1.00 (1.00, 1.00) | 0.1809 |
|  |  |  |  |  |  |  |

A new FI was constructed after excluding platelet counts. And then association of CBC parameters and CBC-derived inflammatory markers with the risk of frailty in middle-aged and elderly people was analyzed. Data are presented as OR (95% CI). Crude adjusts for none. Model 1: adjusted for age, gender, and race; Model 2 was adjusted for model 1 variables plus marital status, education level, family poverty income ratio, body mass index, smoking status, and alcohol status.

**Table S8.** Associations of CBC parameters and CBC-derived inflammatory markers with all-cause mortality in middle-aged and elderly populations with frailty.

|  | Crude |  | Model 1 |  | Model 2 |  |
| --- | --- | --- | --- | --- | --- | --- |
|  | HR (95%CI) | P value | HR (95%CI) | P value | HR (95%CI) | P value |
| NLR |  |  |  |  |  |  |
| Quartile1 | 1(Reference) |  | 1(Reference) |  | 1(Reference) |  |
| Quartile2 | 1.22 (1.08, 1.38) | 0.0014 | 1.07 (0.95, 1.21) | 0.2772 | 1.06 (0.94, 1.19) | 0.3727 |
| Quartile3 | 1.51 (1.35, 1.70) | <0.0001 | 1.22 (1.08, 1.37) | 0.0011 | 1.22 (1.08, 1.37) | 0.0011 |
| Quartile4 | 2.37 (2.12, 2.65) | <0.0001 | 1.73 (1.54, 1.94) | <0.0001 | 1.73 (1.54, 1.95) | <0.0001 |
| P for trend | 1.37 (1.32, 1.42) | <0.0001 | 1.23 (1.19, 1.28) | <0.0001 | 1.24 (1.19, 1.29) | <0.0001 |
| MLR |  |  |  |  |  |  |
| Quartile1 | 1(Reference) |  | 1(Reference) |  | 1(Reference) |  |
| Quartile2 | 1.24 (1.10, 1.41) | 0.0005 | 1.00 (0.88, 1.13) | 0.9967 | 1.03 (0.91, 1.17) | 0.6391 |
| Quartile3 | 1.78 (1.58, 2.01) | <0.0001 | 1.27 (1.13, 1.44) | 0.0001 | 1.33 (1.17, 1.50) | <0.0001 |
| Quartile4 | 2.76 (2.47, 3.09) | <0.0001 | 1.60 (1.42, 1.80) | <0.0001 | 1.69 (1.50, 1.91) | <0.0001 |
| P for trend | 24.39 (17.86, 33.30) | <0.0001 | 5.05 (3.61, 7.07) | <0.0001 | 5.85 (4.18, 8.20) | <0.0001 |
| PLR |  |  |  |  |  |  |
| Quartile1 | 1(Reference) |  | 1(Reference) |  | 1(Reference) |  |
| Quartile2 | 1.01 (0.90, 1.14) | 0.8128 | 0.97 (0.86, 1.09) | 0.5634 | 0.99 (0.88, 1.11) | 0.8703 |
| Quartile3 | 1.08 (0.96, 1.21) | 0.1832 | 0.98 (0.87, 1.10) | 0.7144 | 1.04 (0.92, 1.16) | 0.5474 |
| Quartile4 | 1.47 (1.32, 1.63) | <0.0001 | 1.21 (1.08, 1.34) | 0.0008 | 1.26 (1.13, 1.41) | <0.0001 |
| P for trend | 1.00 (1.00, 1.00) | <0.0001 | 1.00 (1.00, 1.00) | <0.0001 | 1.00 (1.00, 1.00) | <0.0001 |
| SII |  |  |  |  |  |  |
| Quartile1 | 1(Reference) |  | 1(Reference) |  | 1(Reference) |  |
| Quartile2 | 1.22 (1.08, 1.37) | 0.0011 | 1.15 (1.02, 1.30) | 0.0185 | 1.15 (1.02, 1.30) | 0.0182 |
| Quartile3 | 1.28 (1.14, 1.43) | <0.0001 | 1.21 (1.08, 1.36) | 0.0012 | 1.21 (1.08, 1.36) | 0.0015 |
| Quartile4 | 1.69 (1.51, 1.89) | <0.0001 | 1.55 (1.38, 1.73) | <0.0001 | 1.54 (1.37, 1.72) | <0.0001 |
| P for trend | 1.00 (1.00, 1.00) | <0.0001 | 1.00 (1.00, 1.00) | <0.0001 | 1.00 (1.00, 1.00) | <0.0001 |
| SIRI |  |  |  |  |  |  |
| Quartile1 | 1(Reference) |  | 1(Reference) |  | 1(Reference) |  |
| Quartile2 | 1.43 (1.27, 1.61) | <0.0001 | 1.30 (1.15, 1.46) | <0.0001 | 1.25 (1.11, 1.42) | 0.0003 |
| Quartile3 | 1.62 (1.44, 1.81) | <0.0001 | 1.30 (1.15, 1.46) | <0.0001 | 1.28 (1.14, 1.45) | <0.0001 |
| Quartile4 | 2.55 (2.28, 2.86) | <0.0001 | 1.91 (1.70, 2.15) | <0.0001 | 1.87 (1.66, 2.10) | <0.0001 |
| P for trend | 1.56 (1.48, 1.64) | <0.0001 | 1.35 (1.28, 1.43) | <0.0001 | 1.35 (1.28, 1.42) | <0.0001 |
| PIV |  |  |  |  |  |  |
| Quartile1 | 1(Reference) |  | 1(Reference) |  | 1(Reference) |  |
| Quartile2 | 1.15 (1.02, 1.29) | 0.0204 | 1.09 (0.97, 1.22) | 0.1563 | 1.08 (0.96, 1.22) | 0.1936 |
| Quartile3 | 1.33 (1.18, 1.48) | <0.0001 | 1.23 (1.09, 1.38) | 0.0005 | 1.22 (1.09, 1.37) | 0.0008 |
| Quartile4 | 1.78 (1.60, 1.99) | <0.0001 | 1.55 (1.39, 1.74) | <0.0001 | 1.52 (1.36, 1.71) | <0.0001 |
| P for trend | 1.00 (1.00, 1.00) | <0.0001 | 1.00 (1.00, 1.00) | <0.0001 | 1.00 (1.00, 1.00) | <0.0001 |
| Neutrophils |  |  |  |  |  |  |
| Quartile1 | 1(Reference) |  | 1(Reference) |  | 1(Reference) |  |
| Quartile2 | 1.14 (1.02, 1.28) | 0.0191 | 1.04 (0.93, 1.16) | 0.5226 | 1.01 (0.90, 1.13) | 0.8341 |
| Quartile3 | 1.22 (1.09, 1.36) | 0.0007 | 1.14 (1.02, 1.28) | 0.0227 | 1.10 (0.98, 1.23) | 0.1135 |
| Quartile4 | 1.38 (1.23, 1.54) | <0.0001 | 1.50 (1.34, 1.68) | <0.0001 | 1.41 (1.26, 1.58) | <0.0001 |
| P for trend | 1.09 (1.06, 1.12) | <0.0001 | 1.12 (1.09, 1.16) | <0.0001 | 1.11 (1.07, 1.14) | <0.0001 |
| Monocyte |  |  |  |  |  |  |
| Quartile1 | 1(Reference) |  | 1(Reference) |  | 1(Reference) |  |
| Quartile2 | 1.17 (1.04, 1.31) | 0.0097 | 1.10 (0.98, 1.24) | 0.1165 | 1.11 (0.99, 1.25) | 0.0800 |
| Quartile3 | 1.20 (1.07, 1.35) | 0.0020 | 1.08 (0.96, 1.22) | 0.1802 | 1.08 (0.96, 1.22) | 0.1842 |
| Quartile4 | 1.44 (1.30, 1.60) | <0.0001 | 1.26 (1.13, 1.40) | <0.0001 | 1.23 (1.11, 1.37) | <0.0001 |
| P for trend | 2.38 (1.88, 3.01) | <0.0001 | 1.73 (1.35, 2.20) | <0.0001 | 1.62 (1.27, 2.06) | 0.0001 |
| Lymphocyte |  |  |  |  |  |  |
| Quartile1 | 1(Reference) |  | 1(Reference) |  | 1(Reference) |  |
| Quartile2 | 0.69 (0.62, 0.76) | <0.0001 | 0.81 (0.73, 0.90) | <0.0001 | 0.79 (0.71, 0.87) | <0.0001 |
| Quartile3 | 0.52 (0.47, 0.58) | <0.0001 | 0.71 (0.64, 0.79) | <0.0001 | 0.69 (0.62, 0.77) | <0.0001 |
| Quartile4 | 0.47 (0.42, 0.52) | <0.0001 | 0.75 (0.67, 0.84) | <0.0001 | 0.69 (0.62, 0.78) | <0.0001 |
| P for trend | 0.63 (0.59, 0.67) | <0.0001 | 0.83 (0.78, 0.89) | <0.0001 | 0.80 (0.74, 0.85) | <0.0001 |
| Platelet |  |  |  |  |  |  |
| Quartile1 | 1(Reference) |  | 1(Reference) |  | 1(Reference) |  |
| Quartile2 | 0.73 (0.65, 0.81) | <0.0001 | 0.80 (0.72, 0.89) | <0.0001 | 0.82 (0.73, 0.91) | 0.0002 |
| Quartile3 | 0.65 (0.58, 0.72) | <0.0001 | 0.81 (0.73, 0.91) | 0.0002 | 0.79 (0.71, 0.88) | <0.0001 |
| Quartile4 | 0.60 (0.54, 0.67) | <0.0001 | 0.87 (0.77, 0.97) | 0.0115 | 0.85 (0.76, 0.95) | 0.0044 |
| P for trend | 1.00 (1.00, 1.00) | <0.0001 | 1.00 (1.00, 1.00) | 0.0403 | 1.00 (1.00, 1.00) | 0.0097 |
|  |  |  |  |  |  |  |

A new FI was constructed after excluding platelet counts. And then associations of CBC parameters and CBC-derived inflammatory markers with all-cause mortality in middle-aged and elderly populations with frailty. Data are presented as HR (95% CI). Crude adjusts for none. Model 1: adjusted for age, gender, and race; Model 2 was adjusted for model 1 variables plus marital status, education level, family poverty income ratio, body mass index, smoking status, and alcohol status.
